# The Occurrence of the Colistin Resistance Gene *mcr-1* in the Haihe River (China)

**DOI:** 10.3390/ijerph14060576

**Published:** 2017-05-29

**Authors:** Dong Yang, Zhigang Qiu, Zhiqiang Shen, Hong Zhao, Min Jin, Huaying Li, Weili Liu, Jun-Wen Li

**Affiliations:** 1 Department of Environment and Health, Tianjin Institute of Health and Environmental Medicine, Key Laboratory of Risk Assessment and Control for Environment and Food Safety, No.1, Dali Road, Tianjin 300050, China; yangd8611@163.com (D.Y.); zhigangqiu99@gmail.com (Z.Q.); szq922990@126.com (Z.S.); jinminzh@126.com (M.J.); liuweili-002@163.com (W.L.); 2 Animals, Plants and Food Testing Center of Tianjin Exit-Entry Inspection & Quarantine Bureau, Tianjin 300461, China; t0421082@163.com; 3 Tianjin Key Laboratory for Prevention and Control of Occupational and Environmental Hazards, Logistics University of Chinese People’s Armed Police Forces, No. 1, Hui-Zhi Ring Road, Tianjin 300309, China; lihuaying_1988@126.com

**Keywords:** colistin-resistance gene, *mcr-1*, water environments, dissemination, public health

## Abstract

Antibiotic failure is occurring worldwide. In a routine surveillance study on antibiotic resistance genes (ARGs) in natural water bodies, we noted the detection of colistin-resistance gene *mcr-1*, previously identified in *Escherichia coli* and *Klebsiella pneumoniae* isolates from human beings and animals in several countries. The *mcr-1* gene might be present in water environments, because aquatic ecosystems are recognized as reservoirs for antibiotic resistant bacteria (ARB) and ARGs. In this study, a qPCR assay was developed to monitor and quantify the *mcr-1* gene in the Haihe River, China. The results showed that all 18 samples collected from different locations over 6 months along the Haihe River were positive for the *mcr-1* gene, and the highest level of *mcr-1* reached 3.81 × 10^5^ gene copies (GC) per liter of water. This is the first study to quantify *mcr-1* in a natural water system by qPCR. Our findings highlight the potential for this antibiotic resistance determinant to spread extensively, suggesting a significant health and ecological impact.

## 1. Introduction

The discovery of penicillin in the 1920s initiated the era of antibiotics. A wide variety of new antibiotics was introduced in the next few decades. Although they were successful in controlling bacterial infections in the early stage of clinical use, antibiotic resistance was identified shortly thereafter [1]. Resistance has now developed to all antibiotics, including the fluoroquinolones, vancomycin, cephalosporins and carbapenems [1,2]. The cause of this crisis has been attributed to the abuse of antibiotic use in clinical practice and excessive use in agriculture and livestock treatment [1,3]. Up to 90% of the antibiotics are excreted into the environment, where they remove sensitive competitors, leaving resistant bacteria to proliferate and evolve [4]. These bacteria may infect humans via direct or indirect contact, and the infections they cause are harder to control as the antibiotics used to treat them become less effective. Less effective antibiotics result in more antibiotics use, longer treatment time, increased medical costs and higher mortality rates [2], leading into a vicious cycle that drives the evolution of antibiotic resistance and increased antibiotic use. In 2013, a report by the U.S. CDC showed that antibiotic resistance was estimated to cause up to 23,000 deaths in the United States, while the resistance threats are more serious in developing countries [5]. As a result, a global action plan on antibiotic resistance was supported at the World Health Assembly in 2015 [6].

Antibiotic Resistance Genes (ARGs), that are the bedrock for the development and dissemination of bacterial resistance, can be inherited from relatives or acquired from non-relatives through mobile genetic elements such as plasmids, transposons and insertion sequences [7,8]. Mediated by plasmids, the horizontal gene transfer (HGT) of ARGs which can occur widely between different species of bacteria in environments is considered to be the most important mechanism promoting the development of antibiotic resistance [4]. In addition, ARGs carried by transposons and insertion sequences can jump randomly on genomes or plasmids, leading to new or multiple resistances, and even super resistance [9]. Further, the newly formed resistant bacteria can spread and transfer the ARGs through vertical and horizontal transfer, resulting in the persistence of the ARGs in the environment. Thus ARGs are becoming recognized as “easy-to-get, hard-to-lose” pollutants in the environment [10].

Growing evidence suggests that water environments are reservoirs for ARB and ARGs [11]. ARGs encoding resistance to tetracyclines, aminoglycosides, macrolides, chloramphenicol, vancomycin, sulfonamides, trimethoprim and β-lactams have been detected in surface water and ground water, and even in tap water [12,13]. Due to the important position in the food chain and the natural circulation, aquatic ecosystems play a vital role inhuman health. In 2011, the NDM-producing bacteria carrying *bla*_NDM-1_ that were found in public drinking water in New Delhi infected a lot of patients in a short time, attracting global attention [14]. Thus, ARGs harboured by bacteria in the water environment can lead to disease dissemination.

The NDM-producing bacteria were multi-resistant to many groups of frontline antimicrobials, but were sensitive to colistin that remains the last line of defense against infectious [15]. To make matters worse, plasmid-mediated transferable colistin resistance encoded by the *mcr-1* gene was described from patients and animal sources in 2016 [16], which is of great concern all over the world, and then this transmissible gene was identified from *E. coli* and *K. pneumoniae* isolates from animals, foodstuffs and humans in several countries (Denmark, Switzerland, Germany, Laos, Thailand, France, Nigeria, Algeria, Portugal, USA, Bangladesh, Cambodia) [17,18]. In addition, the *mcr-1* gene was identified on a multidrug (trimethoprim, tetracycline, aminoglycoside and sulphonamide) resistant plasmid [19]. Yao et al. reported a carbapenem-resistant and colistin-resistant *E. coli* strain producing NDM-9 and MCR-1 that was isolated from a chicken meat sample [20]. Worryingly, such resistance strains might transfer to human, resulting in untreatable infections [20]. Considering that the ARGs acquired by human pathogens have environmental origin [21], we suspected that the *mcr-1* gene might exist in water environments. Therefore it is necessary to detect and monitor the *mcr-1* gene in water environments.

In the present study, a probe-based qPCR assay for quantitative detection of *mcr-1* was developed and applied for quantitation of the *mcr-1* gene in the Haihe River, which is the largest water system in Northern China and flows through extensive agriculture and livestock areas before flowing into the Bohai Sea. The results of this study may help to track the contamination sources of *mcr-1*.

## 2. Materials and Methods

### 2.1. Specific Primers and Probe Design

Gene-specific primers and a probe were designed based on currently available published sequences of *mcr-1* in GenBank (accession Number: KP347127) with Oligo 7.0 (Supplementary materials Figure S1). Oligonucleotide primers and probe were analyzed for the absence of possible hairpins, secondary structure, and melting temperature. Specificity of the primers and probe were verified in silico by _BLAST_N analysis on the National Center for Biotechnology Information (NCBI) database. All of the primers and probe are listed in Table 1 and were synthesized by Invitrogen Co. (Shanghai, China).

### 2.2. Preparation of Standard Curve

Two *E. coli* strains and two *K. pneumoniae* strains (minimum inhibitory concentration (MIC) of colistin of 8 mg/L) isolated from water samples from the Haihe River were screened for the presence of the *mcr-1* gene by PCR with the primers CLR5-F and CLR5-R as previously described [16]. The two *E. coli* strains (designated Haihe-1 and Haihe-2) were positive for the *mcr-1* gene with 100% sequence identity to the *mcr-1* gene sequence reported by Liu and colleagues [16]. The *K. pneumoniae* strains did not possess *mcr-1* gene. The *E. coli* strains and *K. pneumoniae* strains were used as positive and negative controls, respectively, throughout the study.

The standard curve was constructed using standards prepared as described previously [22,23]. The standard plasmid was constructed from the strain Haihe-1 using a pMD^®^ 19-T Vector Cloning Kit (TAKARA, Dalian, China) and the primers CLR5-F and CLR5-R. Plasmid DNA was purified using a QIAprep^®^ spin Miniprep Kit (Qiagen, Hilden, Germany) according to the manufacturer’s instructions and was quantified with the GeneQuant 1300 instrument (GE Healthcare, Chicago, IL, USA). The DNA sequence was confirmed by direct sequencing using an ABI model 3730 automatic DNA sequencer (ABI, Foster City, CA, USA). One microliter of stock DNA contained 3.9 × 10^6^ gene copies (GC) of the plasmid. Serial 10-fold dilutions of DNA were made in nuclease free water containing 100 ng/µL of tRNA, and aliquots were stored at −80 °C until use. Standard curve was generated using 7.8 × 10^0^ to 7.8 × 10^6^ copies of plasmid DNA. The GC of *mcr-1* was determined based on the standard curve.

### 2.3. qPCR Procedures

The qPCR assay was performed under standard conditions, as indicated by the manufacturer, in a ViiA 7 Dx Real-Time PCR System (ABI Foster City, CA, USA). They were amplified in a 20-µL reaction volume using a FastStart Universal Probe Master Kit (04914058001, Roche, Penzberg, Germany) containing 2 µL of DNA, 10 µL of master mix, 400 nM of each primer (M-F and M-R), and 400 nM of probe (M-Probe) (Table 1). The following PCR program was used for amplification: 2 min at 50 °C, then 10 min at 95 °C, followed by 40 cycles of 95 °C for 15 s and 60 °C for 1 min. All samples were tested three times in separate runs and assayed in triplicate for each run, as were the standard, and positive and negative controls. In addition, the specificity of the qPCR assay was verified in vitro using our local collection of 51 strains including 13 colistin-resistance isolates carrying the *mcr-1* gene (Table 2). The number of GC was defined as the average of the three independent runs data obtained.

### 2.4. Detection of mcr-1 Genes in Haihe River

**Figure 1:**
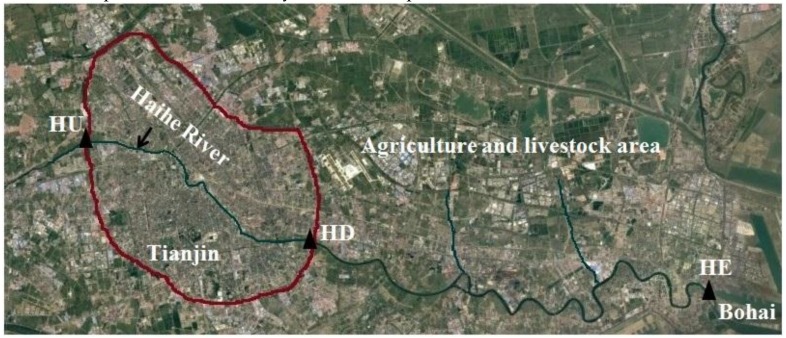
Map showing the sampling locations for the Haihe River (Site HU: upstream of the urban region, 39°10.316’ N, 117°05.395’ E, site HD: downstream of the urban region, 39°01.231’ N, 117°27.344’ E, site HE: estuary of the Haihe River, 39°04.386’ N, 117°18.698’ E).

Samples were collected from the three sites (Figure 1) which were uniformly distributed through the Haihe River. The sampling points HU and HD are located upstream and downstream of the urban regions, respectively, and the HE site was located in the Haihe River estuary (Figure 1). Water samples were collected monthly from sample sites between January and June in 2014. All the samples were collected in sterile containers, transported to the laboratory at 4 °C within 2 h of collection and processed immediately for further experiments.

Methods for the concentration of bacterial cells, were performed as previously described [24]. Briefly, 10 L water samples were filtered through a sterilized steel filter (Millipore, Billerica, MA, U.S.) equipped with a 0.22 μm polycarbonate membrane (100 mm, Millipore, Tokyo, Japan) under 0.15 MPa pressure. Then, the membranes with collected bacteria were immersed in 3% beef extract solution in a magnetic stirring apparatus and incubated for 30 min at 4 °C. The eluted bacteria were recovered by centrifugation at 7000 rpm for 10 min at 4 °C. The pelleted cells were washed three times with phosphate buffered saline (PBS) to eliminate chemical impurities. Finally, the cells were resuspended in 1 mL PBS and stored at −80 °C until use for DNA extraction. Bacterial DNA was extracted using the EZNA Water DNA Kit (Omega Biotek, Norcross, GA, USA) according to the manufacturer’s protocol and the DNA samples with sufficient purity (A260/A280= 1.8–2.0) were applied as template in qPCR analysis. The qPCR assay was then performed using 2 µL of extracted DNA sample.

### 2.5. Statistical Analysis

Data were compiled and statistical tests performed using the Statistical Package for Social Science software (SPSS Inc., Chicago, IL, USA). To evaluate the differences, a two-factor analysis of variance (ANOVA) with model time and site was used on log transformed GC/L data. Post hoc tests were used for multiple comparison, and the Bonferroni method was used in the post hoc tests for site and time. Evaluations were based on *p*-value of <0.05 indicating a significant difference.

## 3. Results

### 3.1. Development of qPCR

A qPCR assay, utilizing a forward primer (M-F primer), a reverse primer (M-R primer), and a probe (M-Probe) was developed. _BLAST_N analysis of the primers and probe showed in silico a 100% homology with MCR-1-encoding gene only (Supplementary Materials Figures S2–S4).

**Figure 2:**
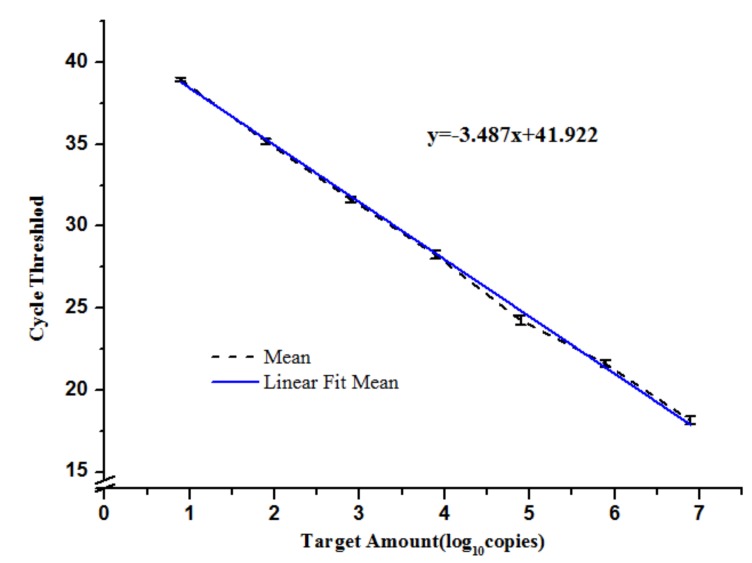
Development of probe-based qPCR: Relationship between known gene copies (GC) number of the standard plasmid DNA and CT values. Average of three reactions is shown and error bars indicate standard deviations.

**Figure 3:**
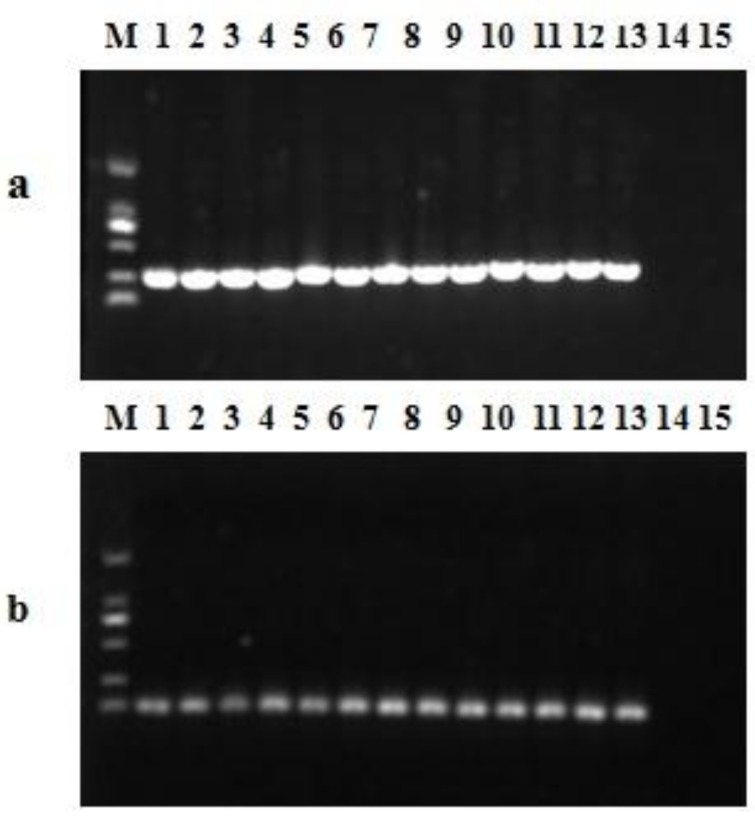
Amplicons of PCR (**a**) and qPCR assay (**b**) from the 51 strains including 13 colistin-resistant isolates carrying the *mcr-1* gene, (**a**) primes CLR5-F and CLR5-R (305bp), lane M: DL 2000 Maker, lanes 1–13 the 13 *mcr-1*-positive strains, lane 14, the *mcr-1*-negative strain, lane 15, no template control; (**b**) qPCR assay (116bp), lane M: DL 2000 Maker, lanes 1–13 the 13 *mcr-1*-positive strains, lane 14, the *mcr-1*-negative strain, lane 15, no template control.

Using a dilution series of 7.8 × 10^0^ to 7.8 × 10^6^ copies of *mcr-1* plasmid DNA, and the lower quantification limit of DNA was equivalent to 8 GC per reaction, indicating that the sensitivity of the qPCR assay was excellent. The crossing points were linearly proportional to the logarithm of the input copy number over 10 orders of magnitude (R^2^ = 0.998). The Y intercept was 41.922, while the slope (S) of the linear regression curve correlates with the efficiency (E) of the PCR reaction according to the formula: E = (10^−1/slope^)–1 [25]. The calculated PCR efficiency for this assay, based on the slope value of −3.487, was 0.94 (Figure 2). The specificity of the qPCR assay in vivo against the 38 *mcr-1*-negative strains was 100% (Table 2). As shown in Figure 3, single products of the 13 *mcr-1*-positive strains were amplified by PCR and qPCR assay.

### 3.2. The mcr-1 Genes in Haihe River

**Figure 4:**
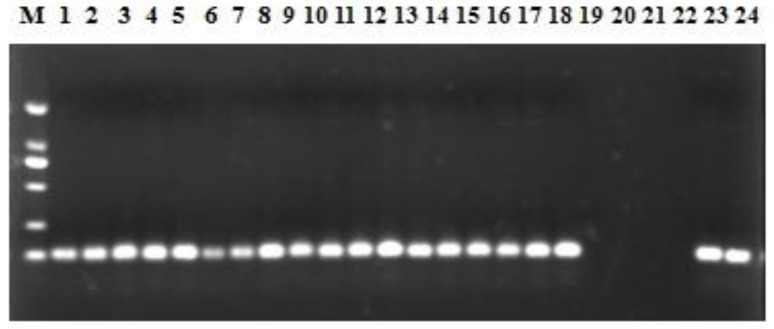
qPCR amplicons (116 bp) from the 18 water samples: lane M, DL 2000 Maker; lanes 1–18 the 18 water samples; lane 19 and 20, the negative control; lane 21 and 22, no template control; lane 23 and 24 the positive control.

**Figure 5:**
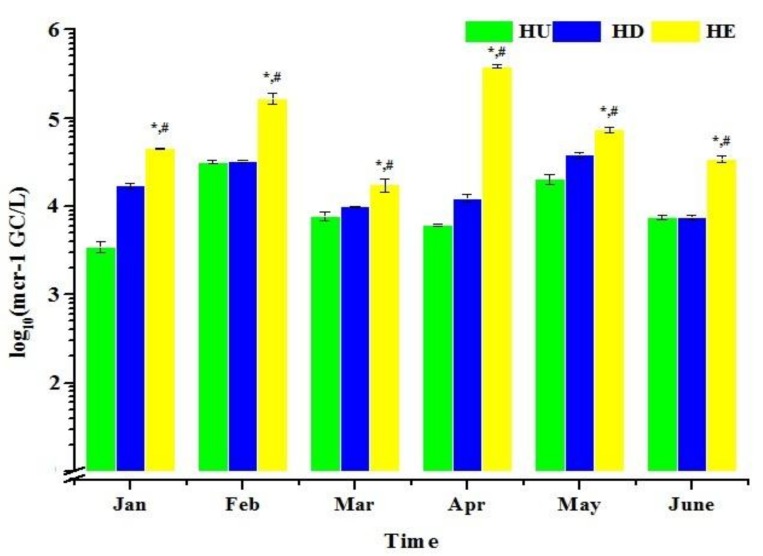
The number of *mcr-1* in water samples. Average of three reactions is shown and error bars indicate standard deviations. The abundance of *mcr-1* was significantly related to site (ANOVA, the abundance of *mcr-1* at site HE have significant different with either HU (*p = 0.001, <0.05) or HD (^#^*p* = 0.011, <0.05) in each month). (Site HU: upstream of the urban region, 39°10.316’ N, 117°05.395’ E, site HD: downstream of the urban region, 39°01.231’ N, 117°27.344’ E, site HE: estuary of the Haihe River, 39°04.386’ N, 117°18.698’ E).

We screened 18 water samples collected during 2014 (between January and June) from the Haihe River for colistin resistance using the qPCR assay developed in the present study. The results showed that all 18 samples were positive for the *mcr-1* (Figure 4). When considering sites individually, the abundance of *mcr-1* gene range from 3.0 × 10^3^ to 3.07 × 10^4^ GC/L, 7.41 × 10^3^ to 3.89 × 10^4^ GC/L and 1.81 × 10^4^ to 3.81 × 10^5^ GC/L at site HU, HD and HE, respectively (Figure 5). The highest *mcr-1* level of the site HE reached 3.81 × 10^5^ GC/L in April. Therefore this discovery implied the presence of *mcr-1* gene in Haihe River as early as 2014. In addition, dilutions of the samples DNA were detected and the relative quantities were not increased, indicating no interference from PCR inhibiting substances.

Analysis of variance (ANOVA) resulting from tests of between-subjects showed no significant differences between time in terms of the abundance of *mcr-1* (*p* = 0.065). However, the abundance of *mcr-1* was significantly related to site (*p* = 0.001, <0.05). The multiple comparisons showed that the abundance of *mcr-1* at site HE have significant different with either HU (*p* < 0.05) or HD (*p* < 0.05) in each month, suggesting that the *mcr-1* was significantly more abundant at site HE than at HU and HD. In addition, there was no significant difference between HU and HD when the abundance of *mcr-1* was considered (*p* = 0.515).

## 4. Discussion

The rise in levels of antibiotic resistance is an urgent global public health concern [26]. Antibiotic resistance poses a serious risk to human health, food safety and social development, which can affect anyone of any age in anywhere in the world [27]. So that, we are entering into a no-antibiotic era, in which resistant bacterial infections can return as an even deadlier threat.

In the past, it was believed that the colistin resistance was only mediated through modulation of two-component regulatory systems resulting in modification of lipid A, which does not seriously affect the use of colistin [28]. Moreover, these resistance genes are generally not transmissible through HGT between bacteria. Thus, the colistin remains the last antibiotic and backbone of defense against multiply resistant Gram-negative bacilli [15]. However, Liu and colleagues described plasmid-mediated colistin resistance involving the *mcr-1* for the first time, which has enormous implications [16]. After the publication of Liu’s report, investigators have shown that similar mechanisms of colistin resistance have been detected in the neighboring southeast Asian countries and even in Europe and Africa [29,30], suggesting that this is already a truly global phenomenon.

The major goal of this study was to develop a probe-based qPCR assay for efficient surveillance and detection of *mcr-1* gene. This goal was accomplished with an assay that was shown to be sensitive and specific for the detection of *mcr-1*. All 13 *mcr-1*-positive samples were detected by our assay and all 38 *mcr-1*-negative samples were negative, showing 100% sensitivity and specificity (Figure 3). In addition, Agarose gel electrophoresis followed the qPCR assay (Figure 4), and the results showed that the amplified products of the water samples were single and sequences were 100% (Supplementary Materials Figure S5) identical to the *mcr-1* gene sequence reported by Liu and colleagues [16]. Recently, Bontron et al. have reported a qPCR assay for detection of *mcr-1* cultured bacteria and stools using SYBR green as fluorescent marker [31]. Chabou et al. set up a qPCR assay with FAM/TAMRA probe for rapid detection of *mcr-1* [25]. It is well known that probes enhance specificity, however, Hong-xia Li reported that the FAM/BHQ was better than the probe FAM /TAMRA under the same fluorescent PCR reaction system [32]. The detection limit of 8 GC reported here is also comparable with the sensitivity of 10^1^ DNA copies reported by Chabou et al. [25].

To the best of our knowledge, this is the first study that quantified the *mcr-1* gene in a natural water system by qPCR, and our results showed that the *mcr-1* gene has been detected along the Haihe River (Figure 4 and Figure 5) which could act as a reservoir of the *mcr-1* gene. The higher abundance of *mcr-1* at HE may result from a combination of potential sources: agriculture, livestock and humans. The Haihe River, one of the seven major rivers, is the largest water system in North China, which may influence the agriculture production and public health [33,34]. Therefore, our results indicated that the *mcr-1* detected in Haihe River should be considered a potential public health risk. In addition, it has been demonstrated that ARGs can transfer between organisms in water environments [35]. More importantly, some reports have shown that many kinds of contaminants in water, such as antibiotics, metals and nanomaterials, can promote the transmission of ARGs [36,37]. Even wastewater treatment processes and drinking water chlorination could significantly affect the efficiency of ARG transfer [38]. The findings highlighted the potential for the *mcr-1* gene to spread extensively, resulting in significant health and ecological impact. Additional studies are needed to identify the *mcr-1* harboring plasmids from the Haihe River and clarify the plasmid backbone. Further, it is necessary to ensure food and environmental safety by law and to curtail the inappropriate prescribing and extensive agricultural use of antibiotics in China.

## 5. Conclusions

A sensitive and specific probe-based qPCR assay was developed for the quantification of the *mcr-1* gene. We successfully deployed the assay in quantification of *mcr-1* in natural water samples taken from the Haihe River and demonstrated an increasing concentration gradient from upstream to downstream through the city of Tianjin and an area with heavy agricultural and livestock presence.

## Figures and Tables

**Table 1:** Primers and probe designed to target the *mcr-1* gene.

Primer/Probe Name	Target Sequences	Sequence (5’–3’)	Tm (°C)	Product Length (bp)	Reference
qPCR	locus region 22541-22635 in the KP347227			116	This study
M-F		CATCGCGGACAATCTCGG	57.2		
M-R		AAATCAACACAGGCTTTAGCAC	55.9		
M-Probe		FAM-AACAGCGTGGTGATCAGTAGCAT-BHQ	61.4		
Standard-PCR	locus region 22447-22755 in the KP347227			305	[16]
CLR5-F		CGGTCAGTCCGTTTGTTC	56.1		
CLR5-R		CTTGGTCGGTCTGTAGGG	56.3		

**Table 2:** Presentation of the specificity of the qPCR assay.

Species	Presence of *mcr-1* Gene	COL MIC(mg/L)	CT Value	Origins
*E.coli* (*n* = 11)	+	4–16	15–28	China (water environment)
*E.coli* (*n* = 7)	−	<1	<LOQ	
*K.pneumoniae* (*n* = 2)	+	8	18–28	
*K.pneumoniae* (*n* = 10)	−	<1	<LOQ	
*Morganellamorganii* (*n* = 3)	−	>256	<LOQ	
*Providenciaalcalifaciens* (*n* = 5)	−	<1	<LOQ	
*Serratiamarcescens* (*n* = 4)	−	>128	<LOQ	
*Yokenellaregensburgei* (*n* = 5)	−	>128	<LOQ	
*Aeromonashydrophila *(*n* = 4)	−	>128	<LOQ	

+ positive; − negative; COL MIC, minimum inhibitory concentration of colistin; LOQ, Limit of Quantity.

## Supplementary Materials

The following are available online at http://www.mdpi.com/1660-4601/14/6/576/s1, Figure S1: The scores of product, prime and probe in Oligo 7.0, Figure S2: _BLAST_N analysis of primer M-F, Figure S3: _BLAST_N analysis of primer M-R, Figure S4:_ BLAST_N analysis of primer M-Probe, Figure S5: Alignment of the sequences of qPCR products and the *mcr-1* gene sequence: (KP347127: the *mcr-1* gene sequence reported by Liu and colleagues, s-P: positive control, s-1–s-18: water samples), Figure S6: Recovery of Haihe-1 strain (10^1^ CFU/L–10^7^ CFU/L) spiked into 10 L PBS, Figure S7: Recovery of Haihe-1 strain (10^1^ CFU/L–10^7^ CFU/L) spiked into 10 L sterilized waster sample, Figure S8: The deviance analysis result of ANOVA, Figure S9: The multiple comparisons result of ANOVA.
