# Supplementary material for: The Occurrence of the Colistin Resistance Gene mcr-1 in the Haihe River (China)
Source: Int J Environ Res Public Health. 2017 May 29;14(6):576. doi: 10.3390/ijerph14060576 (PMC5486262; doi:10.3390/ijerph14060576)
Supplement: Supplementary file 1 [file ijerph-14-00576-s001.pdf]

International Journal of
Environmental Research and Public Health

MDPI

Supplementary Materials: The Occurrence of the Colistin Resistance Gene *mcr-1* in the Haihe River (China)

Dong Yang [1,†], Zhigang Qiu [1,†], Zhiqiang Shen [1,†], Hong Zhao [2], Min Jin [1], Huaying Li [3], Weili Liu [1] and Jun-Wen Li [1,*]

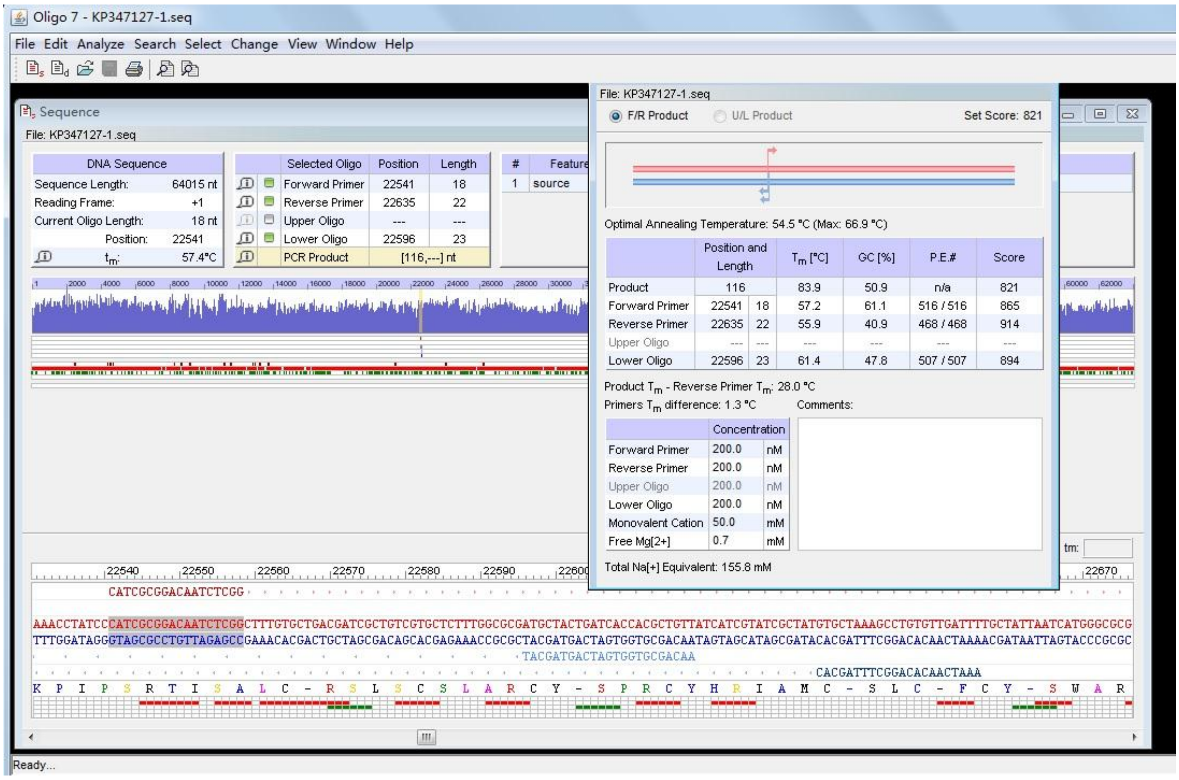

Figure S1. The scores of product, prime and probe in Oligo 7.0.

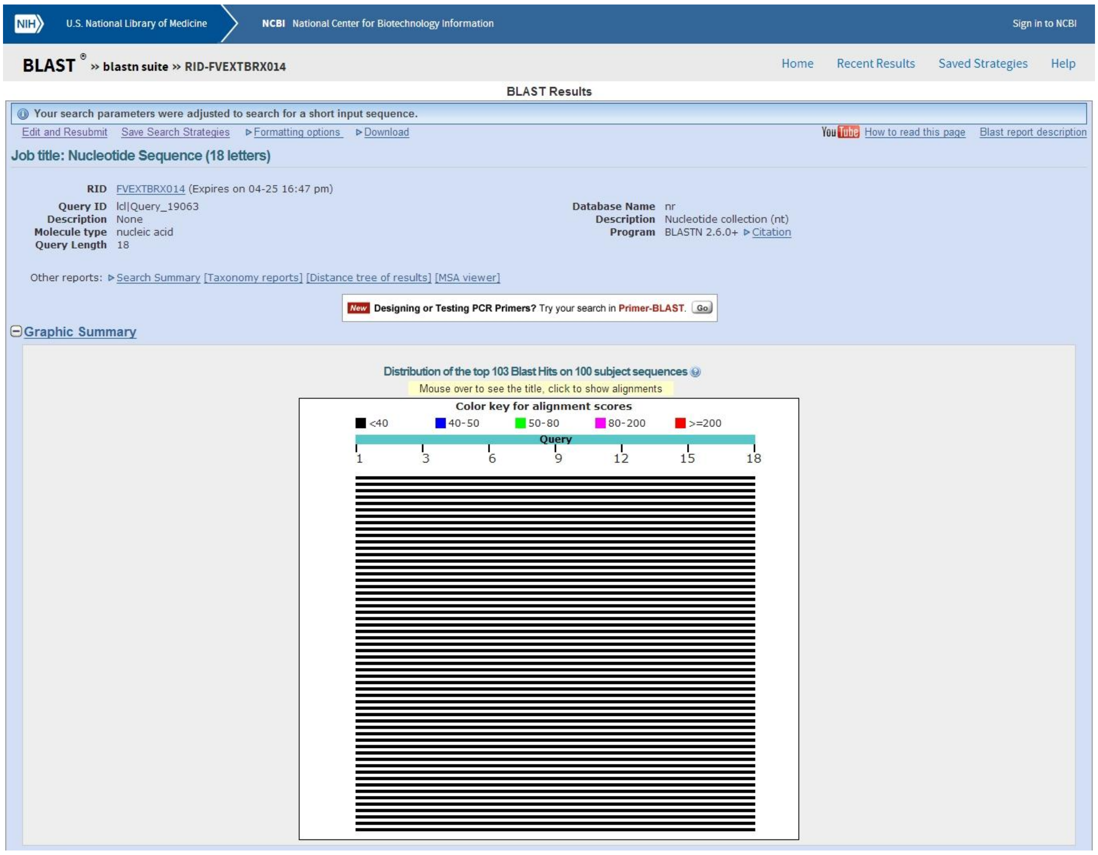

Figure S2. *Cont.*

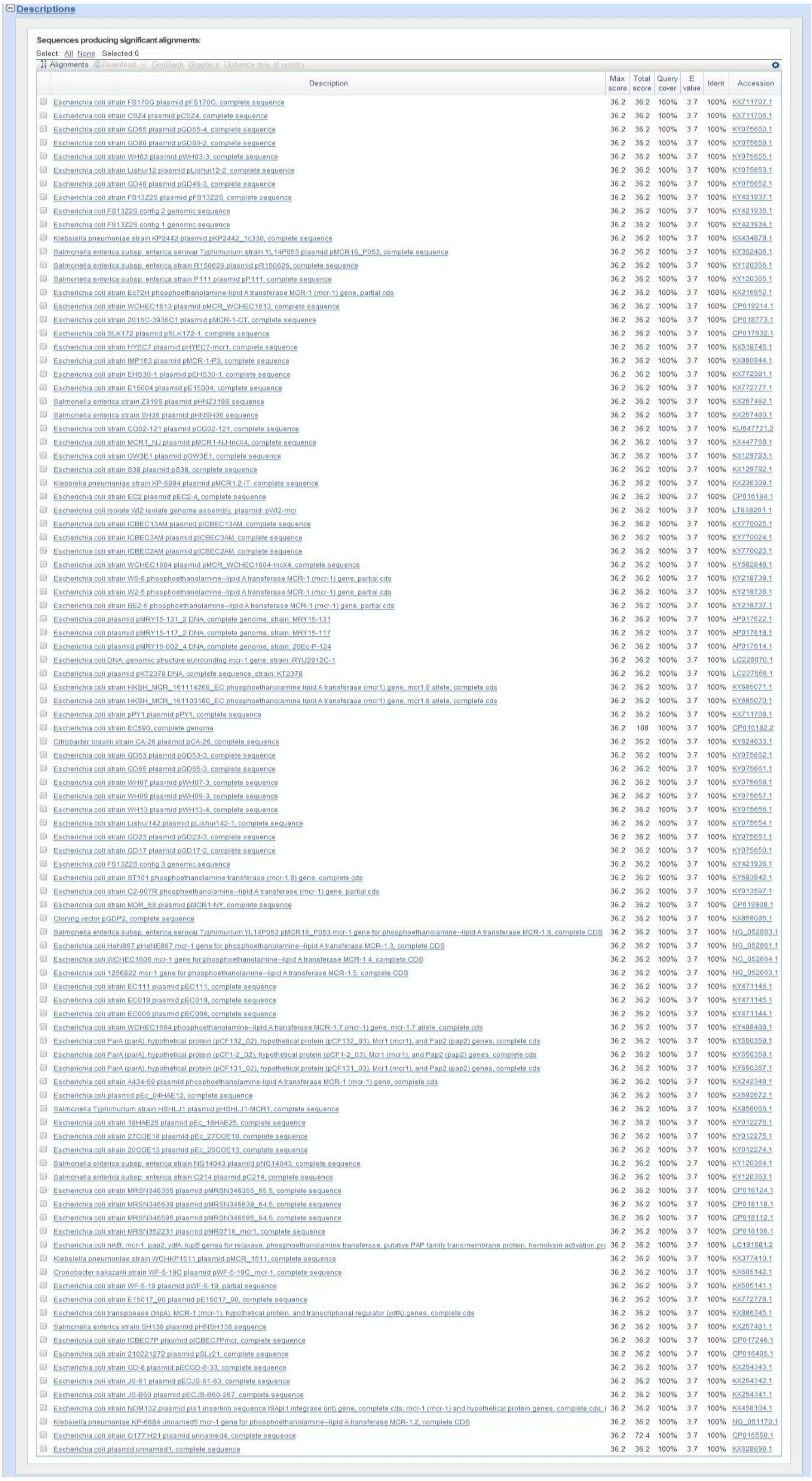

Descriptions

Sequences producing significant alignments:

Select: All None Selected:0

Alignments Download GenBank Graphics Distance tree of results

Description	Max score	Total score	Query cover	E value	Ident	Accession
Escherichia coli strain FS170G plasmid pFS170G, complete sequence	36.2	36.2	100%	3.7	100%	KX711707.1
Escherichia coli strain CSZ4 plasmid pCSZ4, complete sequence	36.2	36.2	100%	3.7	100%	KX711706.1
Escherichia coli strain GD65 plasmid pGD65-4, complete sequence	36.2	36.2	100%	3.7	100%	KY075660.1
Escherichia coli strain GD80 plasmid pGD80-2, complete sequence	36.2	36.2	100%	3.7	100%	KY075659.1
Escherichia coli strain WH03 plasmid pWH03-3, complete sequence	36.2	36.2	100%	3.7	100%	KY075655.1
Escherichia coli strain Lishui12 plasmid pLishui12-2, complete sequence	36.2	36.2	100%	3.7	100%	KY075653.1
Escherichia coli strain GD46 plasmid pGD46-3, complete sequence	36.2	36.2	100%	3.7	100%	KY075652.1
Escherichia coli strain FS13Z2S plasmid pFS13Z2S, complete sequence	36.2	36.2	100%	3.7	100%	KY421937.1
Escherichia coli FS13Z2S contig 2 genomic sequence	36.2	36.2	100%	3.7	100%	KY421935.1
Escherichia coli FS13Z2S contig 1 genomic sequence	36.2	36.2	100%	3.7	100%	KY421934.1
Klebsiella pneumoniae strain KP2442 plasmid pKP2442_1c330, complete sequence	36.2	36.2	100%	3.7	100%	KX434879.1
Salmonella enterica subsp. enterica serovar Typhimurium strain YL14P053 plasmid pMCR16_P053, complete sequence	36.2	36.2	100%	3.7	100%	KY352406.1
Salmonella enterica subsp. enterica strain R150626 plasmid pR150626, complete sequence	36.2	36.2	100%	3.7	100%	KY120366.1
Salmonella enterica subsp. enterica strain P111 plasmid pP111, complete sequence	36.2	36.2	100%	3.7	100%	KY120365.1
Escherichia coli strain Ec72H phosphoethanolamine-lipid A transferase MCR-1 (mcr-1) gene, partial cds	36.2	36.2	100%	3.7	100%	KX216852.1
Escherichia coli strain WCHEC1613 plasmid pMCR_WCHEC1613, complete sequence	36.2	36.2	100%	3.7	100%	CP019214.1
Escherichia coli strain 2016C-3936C1 plasmid pMCR-1-CT, complete sequence	36.2	36.2	100%	3.7	100%	CP018773.1
Escherichia coli SLK172 plasmid pSLK172-1, complete sequence	36.2	36.2	100%	3.7	100%	CP017632.1
Escherichia coli strain HYEC7 plasmid pHYEC7-mcr1, complete sequence	36.2	36.2	100%	3.7	100%	KX518745.1
Escherichia coli strain IMP163 plasmid pMCR-1-P3, complete sequence	36.2	36.2	100%	3.7	100%	KX880944.1
Escherichia coli strain EHS30-1 plasmid pEHS30-1, complete sequence	36.2	36.2	100%	3.7	100%	KX772391.1
Escherichia coli strain E15004 plasmid pE15004, complete sequence	36.2	36.2	100%	3.7	100%	KX772777.1
Salmonella enterica strain Z319S plasmid pHNZ319S sequence	36.2	36.2	100%	3.7	100%	KX257482.1
Salmonella enterica strain SH36 plasmid pHNSH36 sequence	36.2	36.2	100%	3.7	100%	KX257480.1
Escherichia coli strain CQ02-121 plasmid pCQ02-121, complete sequence	36.2	36.2	100%	3.7	100%	KU647721.2
Escherichia coli strain MCR1_NJ plasmid pMCR1-NJ-IncX4, complete sequence	36.2	36.2	100%	3.7	100%	KX447768.1
Escherichia coli strain OW3E1 plasmid pOW3E1, complete sequence	36.2	36.2	100%	3.7	100%	KX129783.1
Escherichia coli strain S38 plasmid pS38, complete sequence	36.2	36.2	100%	3.7	100%	KX129782.1
Klebsiella pneumoniae strain KP-6884 plasmid pMCR1.2-IT, complete sequence	36.2	36.2	100%	3.7	100%	KX236309.1
Escherichia coli strain EC2 plasmid pEC2-4, complete sequence	36.2	36.2	100%	3.7	100%	CP016184.1
Escherichia coli isolate WI2 isolate genome assembly, plasmid: pWI2-mcr	36.2	36.2	100%	3.7	100%	LT838201.1
Escherichia coli strain ICBEC13AM plasmid pICBEC13AM, complete sequence	36.2	36.2	100%	3.7	100%	KY770025.1
Escherichia coli strain ICBEC3AM plasmid pICBEC3AM, complete sequence	36.2	36.2	100%	3.7	100%	KY770024.1
Escherichia coli strain ICBEC2AM plasmid pICBEC2AM, complete sequence	36.2	36.2	100%	3.7	100%	KY770023.1
Escherichia coli strain WCHEC1604 plasmid pMCR_WCHEC1604-IncX4, complete sequence	36.2	36.2	100%	3.7	100%	KY582848.1
Escherichia coli strain W5-6 phosphoethanolamine--lipid A transferase MCR-1 (mcr-1) gene, partial cds	36.2	36.2	100%	3.7	100%	KY218739.1
Escherichia coli strain W2-5 phosphoethanolamine--lipid A transferase MCR-1 (mcr-1) gene, partial cds	36.2	36.2	100%	3.7	100%	KY218738.1
Escherichia coli strain BE2-5 phosphoethanolamine--lipid A transferase MCR-1 (mcr-1) gene, partial cds	36.2	36.2	100%	3.7	100%	KY218737.1
Escherichia coli plasmid pMRY15-131_2 DNA, complete genome, strain: MRY15-131	36.2	36.2	100%	3.7	100%	AP017622.1
Escherichia coli plasmid pMRY15-117_2 DNA, complete genome, strain: MRY15-117	36.2	36.2	100%	3.7	100%	AP017619.1
Escherichia coli plasmid pMRY16-002_4 DNA, complete genome, strain: 20Ec-P-124	36.2	36.2	100%	3.7	100%	AP017614.1
Escherichia coli DNA, genomic structure surrounding mcr-1 gene, strain: RYU2912C-1	36.2	36.2	100%	3.7	100%	LC228070.1
Escherichia coli plasmid pKT2378 DNA, complete sequence, strain: KT2378	36.2	36.2	100%	3.7	100%	LC227558.1
Escherichia coli strain HKSH_MCR_161114268_EC phosphoethanolamine lipid A transferase (mcr1) gene, mcr1.9 allele, complete cds	36.2	36.2	100%	3.7	100%	KY685071.1
Escherichia coli strain HKSH_MCR_161103180_EC phosphoethanolamine lipid A transferase (mcr1) gene, mcr1.8 allele, complete cds	36.2	36.2	100%	3.7	100%	KY685070.1
Escherichia coli strain pPY1 plasmid pPY1, complete sequence	36.2	36.2	100%	3.7	100%	KX711708.1
Escherichia coli strain EC590, complete genome	36.2	108	100%	3.7	100%	CP016182.2
Citrobacter braakii strain CA-26 plasmid pCA-26, complete sequence	36.2	36.2	100%	3.7	100%	KY624633.1
Escherichia coli strain GD53 plasmid pGD53-3, complete sequence	36.2	36.2	100%	3.7	100%	KY075662.1
Escherichia coli strain GD65 plasmid pGD65-3, complete sequence	36.2	36.2	100%	3.7	100%	KY075661.1
Escherichia coli strain WH07 plasmid pWH07-3, complete sequence	36.2	36.2	100%	3.7	100%	KY075658.1
Escherichia coli strain WH09 plasmid pWH09-3, complete sequence	36.2	36.2	100%	3.7	100%	KY075657.1
Escherichia coli strain WH13 plasmid pWH13-4, complete sequence	36.2	36.2	100%	3.7	100%	KY075656.1
Escherichia coli strain Lishui142 plasmid pLishui142-1, complete sequence	36.2	36.2	100%	3.7	100%	KY075654.1
Escherichia coli strain GD23 plasmid pGD23-3, complete sequence	36.2	36.2	100%	3.7	100%	KY075651.1
Escherichia coli strain GD17 plasmid pGD17-2, complete sequence	36.2	36.2	100%	3.7	100%	KY075650.1
Escherichia coli FS13Z2S contig 3 genomic sequence	36.2	36.2	100%	3.7	100%	KY421936.1
Escherichia coli strain ST101 phosphoethanolamine transferase (mcr-1.8) gene, complete cds	36.2	36.2	100%	3.7	100%	KY683842.1
Escherichia coli strain C2-007R phosphoethanolamine--lipid A transferase (mcr-1) gene, partial cds	36.2	36.2	100%	3.7	100%	KY013597.1
Escherichia coli strain MDR_56 plasmid pMCR1-NY, complete sequence	36.2	36.2	100%	3.7	100%	CP019908.1
Cloning vector pGDP2, complete sequence	36.2	36.2	100%	3.7	100%	KX859085.1
Salmonella enterica subsp. enterica serovar Typhimurium YL14P053 pMCR16_P053 mcr-1 gene for phosphoethanolamine--lipid A transferase MCR-1.6, complete CDS	36.2	36.2	100%	3.7	100%	NG_052893.1
Escherichia coli HeN867 pHeNE867 mcr-1 gene for phosphoethanolamine--lipid A transferase MCR-1.3, complete CDS	36.2	36.2	100%	3.7	100%	NG_052861.1
Escherichia coli WCHEC1606 mcr-1 gene for phosphoethanolamine--lipid A transferase MCR-1.4, complete CDS	36.2	36.2	100%	3.7	100%	NG_052664.1
Escherichia coli 1256822 mcr-1 gene for phosphoethanolamine--lipid A transferase MCR-1.5, complete CDS	36.2	36.2	100%	3.7	100%	NG_052663.1
Escherichia coli strain EC111 plasmid pEC111, complete sequence	36.2	36.2	100%	3.7	100%	KY471146.1
Escherichia coli strain EC019 plasmid pEC019, complete sequence	36.2	36.2	100%	3.7	100%	KY471145.1
Escherichia coli strain EC006 plasmid pEC006, complete sequence	36.2	36.2	100%	3.7	100%	KY471144.1
Escherichia coli strain WCHEC1604 phosphoethanolamine--lipid A transferase MCR-1.7 (mcr-1) gene, mcr-1.7 allele, complete cds	36.2	36.2	100%	3.7	100%	KY488488.1
Escherichia coli ParA (parA), hypothetical protein (pCF132_02), hypothetical protein (pCF132_03), Mcr1 (mcr1), and Pap2 (pap2) genes, complete cds	36.2	36.2	100%	3.7	100%	KY550359.1
Escherichia coli ParA (parA), hypothetical protein (pCF1-2_02), hypothetical protein (pCF1-2_03), Mcr1 (mcr1), and Pap2 (pap2) genes, complete cds	36.2	36.2	100%	3.7	100%	KY550358.1
Escherichia coli ParA (parA), hypothetical protein (pCF131_02), hypothetical protein (pCF131_03), Mcr1 (mcr1), and Pap2 (pap2) genes, complete cds	36.2	36.2	100%	3.7	100%	KY550357.1
Escherichia coli strain A434-59 plasmid phosphoethanolamine-lipid A transferase MCR-1 (mcr-1) gene, complete cds	36.2	36.2	100%	3.7	100%	KX242348.1
Escherichia coli plasmid pEc_04HAE12, complete sequence	36.2	36.2	100%	3.7	100%	KX592672.1
Salmonella Typhimurium strain HSHLJ1 plasmid pHSHLJ1-MCR1, complete sequence	36.2	36.2	100%	3.7	100%	KX856066.1
Escherichia coli strain 18HAE25 plasmid pEc_18HAE25, complete sequence	36.2	36.2	100%	3.7	100%	KY012276.1
Escherichia coli strain 27COE18 plasmid pEc_27COE18, complete sequence	36.2	36.2	100%	3.7	100%	KY012275.1
Escherichia coli strain 20COE13 plasmid pEc_20COE13, complete sequence	36.2	36.2	100%	3.7	100%	KY012274.1
Salmonella enterica subsp. enterica strain NG14043 plasmid pNG14043, complete sequence	36.2	36.2	100%	3.7	100%	KY120364.1
Salmonella enterica subsp. enterica strain C214 plasmid pC214, complete sequence	36.2	36.2	100%	3.7	100%	KY120363.1
Escherichia coli strain MRSN346355 plasmid pMRSN346355_65.5, complete sequence	36.2	36.2	100%	3.7	100%	CP018124.1
Escherichia coli strain MRSN346638 plasmid pMRSN346638_64.5, complete sequence	36.2	36.2	100%	3.7	100%	CP018118.1
Escherichia coli strain MRSN346595 plasmid pMRSN346595_64.5, complete sequence	36.2	36.2	100%	3.7	100%	CP018112.1
Escherichia coli strain MRSN352231 plasmid pMR0716_mcr1, complete sequence	36.2	36.2	100%	3.7	100%	CP018106.1
Escherichia coli nikB, mcr-1, pap2, ydfA, topB genes for relaxase, phosphoethanolamine transferase, putative PAP family transmembrane protein, hemolysin activation pr	36.2	36.2	100%	3.7	100%	LC191581.2
Klebsiella pneumoniae strain WCHKP1511 plasmid pMCR_1511, complete sequence	36.2	36.2	100%	3.7	100%	KX377410.1
Cronobacter sakazakii strain WF-5-19C plasmid pWF-5-19C_mcr-1, complete sequence	36.2	36.2	100%	3.7	100%	KX505142.1
Escherichia coli strain WF-5-19 plasmid pWF-5-19, partial sequence	36.2	36.2	100%	3.7	100%	KX505141.1
Escherichia coli strain E15017_00 plasmid pE15017_00, complete sequence	36.2	36.2	100%	3.7	100%	KX772778.1
Escherichia coli transposase (tnpA), MCR-1 (mcr-1), hypothetical protein, and transcriptional regulator (ydfA) genes, complete cds	36.2	36.2	100%	3.7	100%	KX886345.1
Salmonella enterica strain SH138 plasmid pHNSH138 sequence	36.2	36.2	100%	3.7	100%	KX257481.1
Escherichia coli strain ICBEC7P plasmid pICBEC7Pmcr, complete sequence	36.2	36.2	100%	3.7	100%	CP017246.1
Escherichia coli strain 210221272 plasmid pSLy21, complete sequence	36.2	36.2	100%	3.7	100%	CP016405.1
Escherichia coli strain GD-8 plasmid pECGD-8-33, complete sequence	36.2	36.2	100%	3.7	100%	KX254343.1
Escherichia coli strain JS-61 plasmid pECJS-61-63, complete sequence	36.2	36.2	100%	3.7	100%	KX254342.1
Escherichia coli strain JS-B60 plasmid pECJS-B60-267, complete sequence	36.2	36.2	100%	3.7	100%	KX254341.1
Escherichia coli strain NDM132 plasmid pIs1 insertion sequence ISApl1 integrase (int) gene, complete cds; mcr-1 (mcr-1) and hypothetical protein genes, complete cds;	36.2	36.2	100%	3.7	100%	KX458104.1
Klebsiella pneumoniae KP-6884 unnamed5 mcr-1 gene for phosphoethanolamine--lipid A transferase MCR-1.2, complete CDS	36.2	36.2	100%	3.7	100%	NG_051170.1
Escherichia coli strain O177:H21 plasmid unnamed4, complete sequence	36.2	72.4	100%	3.7	100%	CP016550.1
Escherichia coli plasmid unnamed1, complete sequence	36.2	36.2	100%	3.7	100%	KX528699.1

Figure S2. *Cont.*

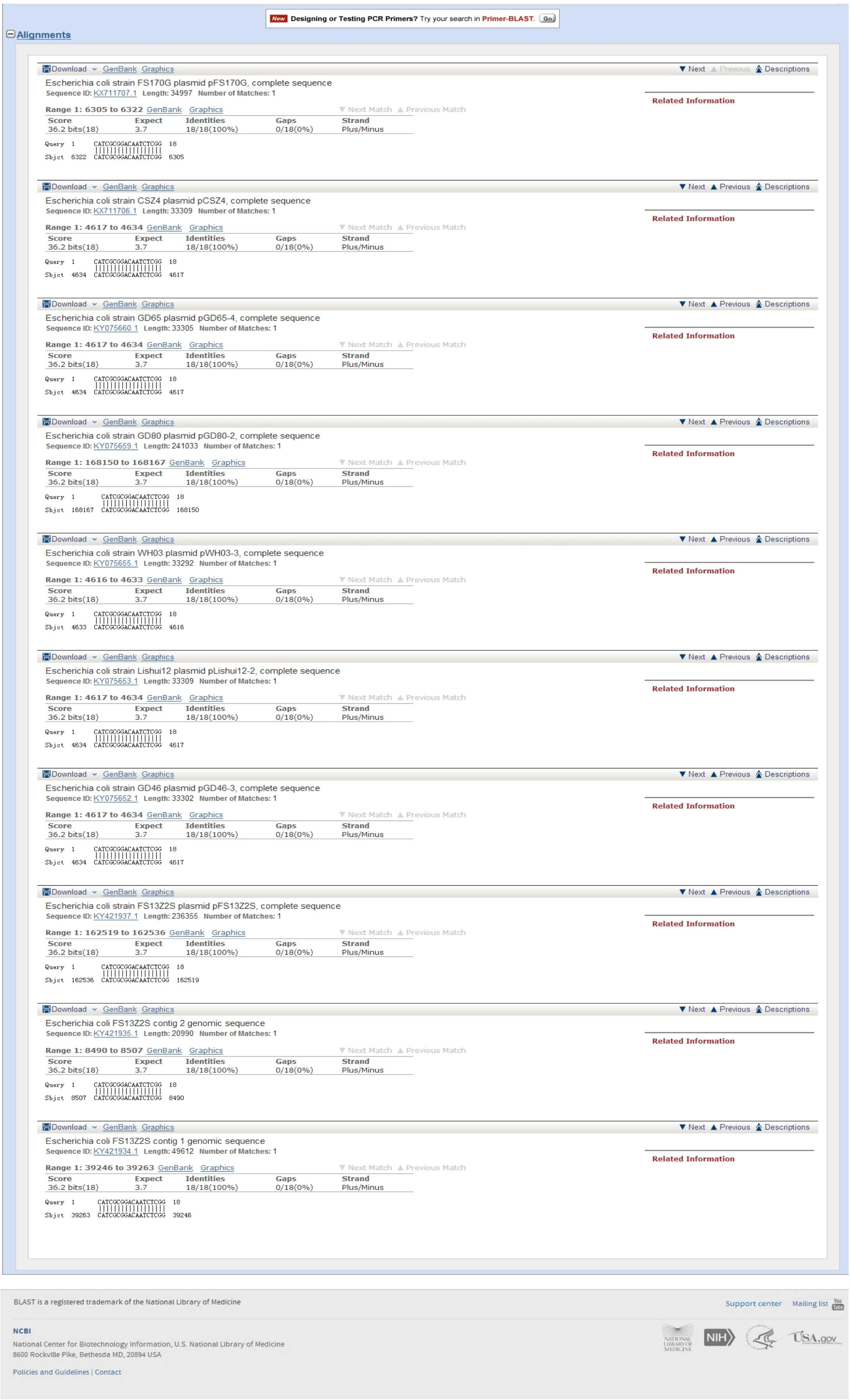

Figure S2. BLASTN analysis of primer M-F.

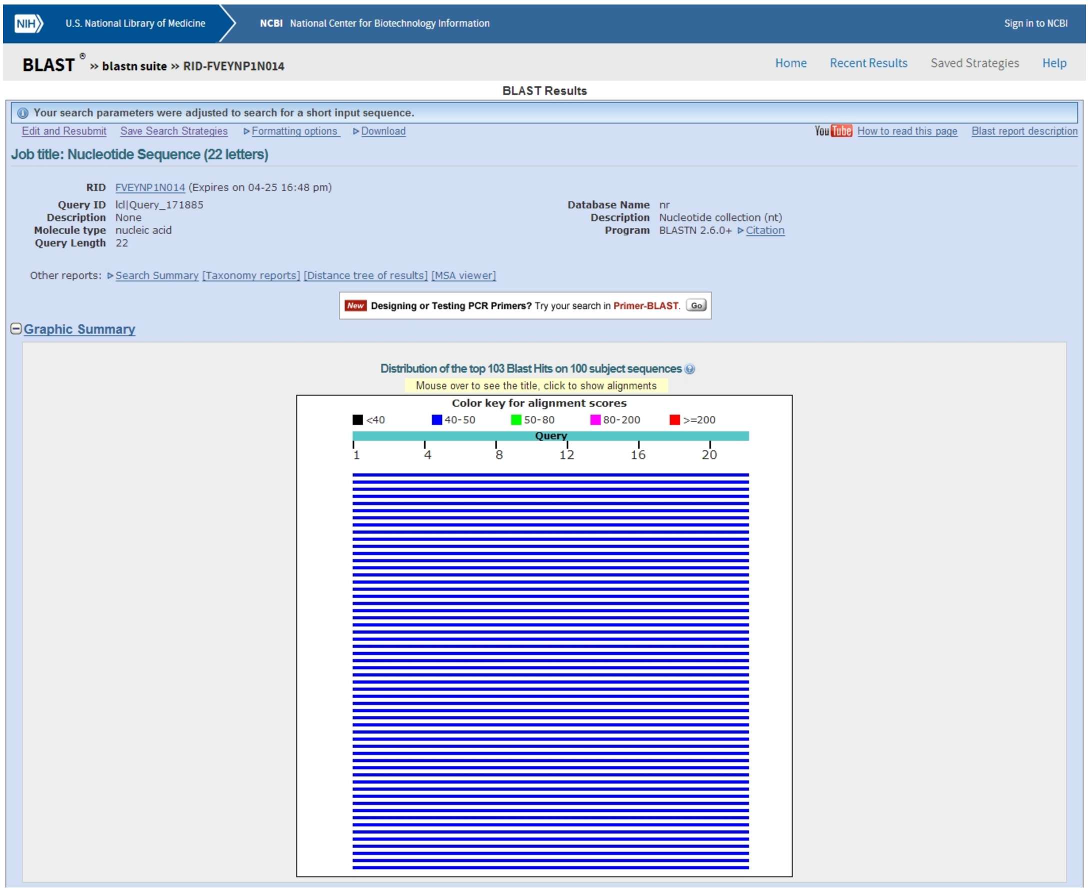

Figure S3. *Cont.*

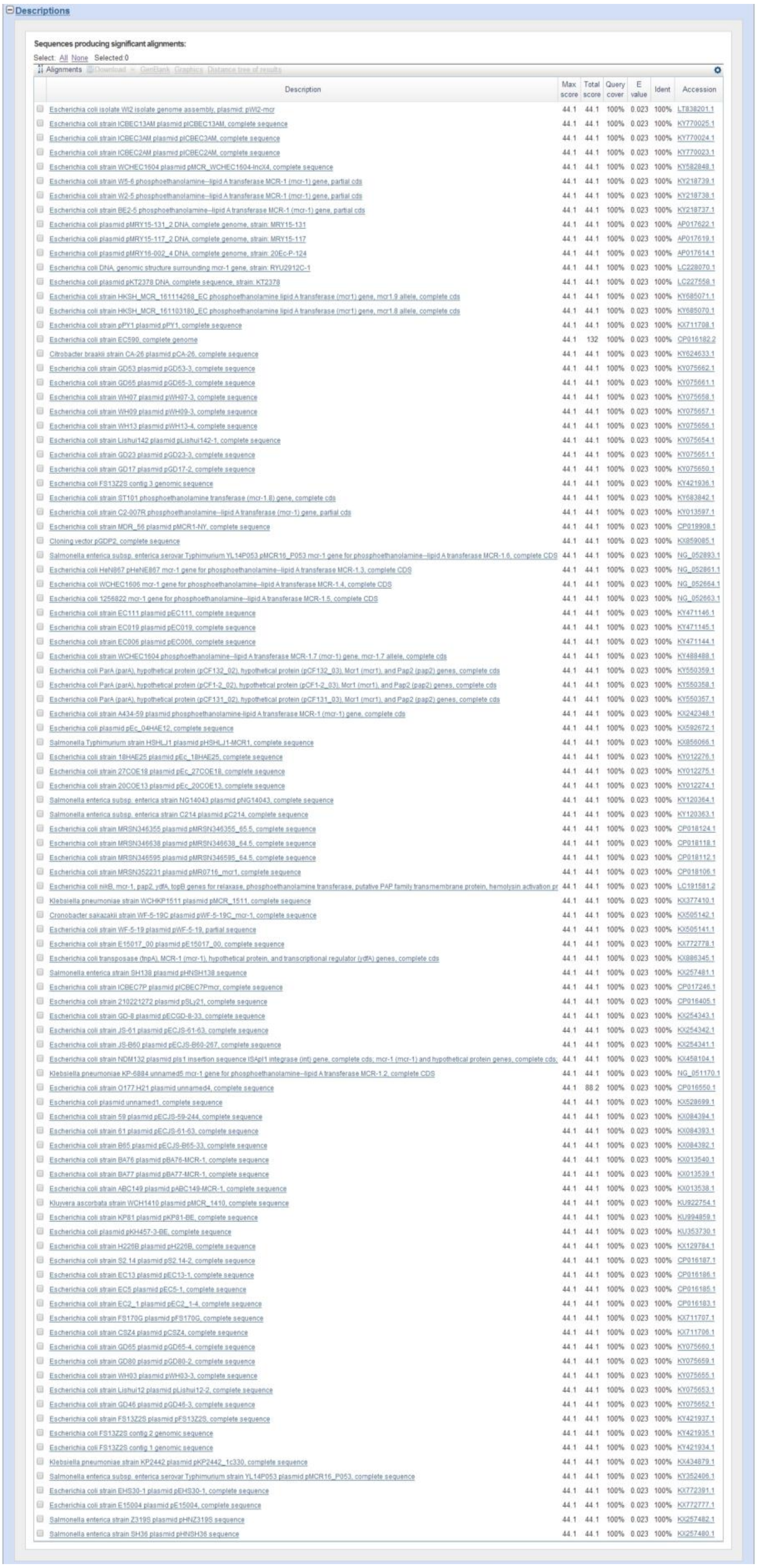

Descriptions

Sequences producing significant alignments:

Select: All None Selected:0

Alignments Download GenBank Graphics Distance tree of results

Description	Max score	Total score	Query cover	E value	Ident	Accession
Escherichia coli isolate WI2 isolate genome assembly, plasmid: pWI2-mcr	44.1	44.1	100%	0.023	100%	LT838201.1
Escherichia coli strain ICBEC13AM plasmid pICBEC13AM, complete sequence	44.1	44.1	100%	0.023	100%	KY770025.1
Escherichia coli strain ICBEC3AM plasmid pICBEC3AM, complete sequence	44.1	44.1	100%	0.023	100%	KY770024.1
Escherichia coli strain ICBEC2AM plasmid pICBEC2AM, complete sequence	44.1	44.1	100%	0.023	100%	KY770023.1
Escherichia coli strain WCHEC1604 plasmid pMCR_WCHEC1604-IncX4, complete sequence	44.1	44.1	100%	0.023	100%	KY582848.1
Escherichia coli strain W5-6 phosphoethanolamine--lipid A transferase MCR-1 (mcr-1) gene, partial cds	44.1	44.1	100%	0.023	100%	KY218739.1
Escherichia coli strain W2-5 phosphoethanolamine--lipid A transferase MCR-1 (mcr-1) gene, partial cds	44.1	44.1	100%	0.023	100%	KY218738.1
Escherichia coli strain BE2-5 phosphoethanolamine--lipid A transferase MCR-1 (mcr-1) gene, partial cds	44.1	44.1	100%	0.023	100%	KY218737.1
Escherichia coli plasmid pMRY15-131_2 DNA, complete genome, strain: MRY15-131	44.1	44.1	100%	0.023	100%	AP017622.1
Escherichia coli plasmid pMRY15-117_2 DNA, complete genome, strain: MRY15-117	44.1	44.1	100%	0.023	100%	AP017619.1
Escherichia coli plasmid pMRY16-002_4 DNA, complete genome, strain: 20Ec-P-124	44.1	44.1	100%	0.023	100%	AP017614.1
Escherichia coli DNA, genomic structure surrounding mcr-1 gene, strain: RYU2912C-1	44.1	44.1	100%	0.023	100%	LC228070.1
Escherichia coli plasmid pKT2378 DNA, complete sequence, strain: KT2378	44.1	44.1	100%	0.023	100%	LC227558.1
Escherichia coli strain HKSH_MCR_161114268_EC phosphoethanolamine lipid A transferase (mcr1) gene, mcr1.9 allele, complete cds	44.1	44.1	100%	0.023	100%	KY685071.1
Escherichia coli strain HKSH_MCR_161103180_EC phosphoethanolamine lipid A transferase (mcr1) gene, mcr1.8 allele, complete cds	44.1	44.1	100%	0.023	100%	KY685070.1
Escherichia coli strain pPY1 plasmid pPY1, complete sequence	44.1	44.1	100%	0.023	100%	KX711708.1
Escherichia coli strain EC590, complete genome	44.1	132	100%	0.023	100%	CP016182.2
Citrobacter braakii strain CA-26 plasmid pCA-26, complete sequence	44.1	44.1	100%	0.023	100%	KY624633.1
Escherichia coli strain GD53 plasmid pGD53-3, complete sequence	44.1	44.1	100%	0.023	100%	KY075662.1
Escherichia coli strain GD65 plasmid pGD65-3, complete sequence	44.1	44.1	100%	0.023	100%	KY075661.1
Escherichia coli strain WH07 plasmid pWH07-3, complete sequence	44.1	44.1	100%	0.023	100%	KY075658.1
Escherichia coli strain WH09 plasmid pWH09-3, complete sequence	44.1	44.1	100%	0.023	100%	KY075657.1
Escherichia coli strain WH13 plasmid pWH13-4, complete sequence	44.1	44.1	100%	0.023	100%	KY075656.1
Escherichia coli strain Lishui142 plasmid pLishui142-1, complete sequence	44.1	44.1	100%	0.023	100%	KY075654.1
Escherichia coli strain GD23 plasmid pGD23-3, complete sequence	44.1	44.1	100%	0.023	100%	KY075651.1
Escherichia coli strain GD17 plasmid pGD17-2, complete sequence	44.1	44.1	100%	0.023	100%	KY075650.1
Escherichia coli FS13Z2S contig 3 genomic sequence	44.1	44.1	100%	0.023	100%	KY421936.1
Escherichia coli strain ST101 phosphoethanolamine transferase (mcr-1.8) gene, complete cds	44.1	44.1	100%	0.023	100%	KY683842.1
Escherichia coli strain C2-007R phosphoethanolamine--lipid A transferase (mcr-1) gene, partial cds	44.1	44.1	100%	0.023	100%	KY013597.1
Escherichia coli strain MDR_56 plasmid pMCR1-NY, complete sequence	44.1	44.1	100%	0.023	100%	CP019908.1
Cloning vector pGDP2, complete sequence	44.1	44.1	100%	0.023	100%	KX859085.1
Salmonella enterica subsp. enterica serovar Typhimurium YL14P053 pMCR16_P053 mcr-1 gene for phosphoethanolamine--lipid A transferase MCR-1.6, complete CDS	44.1	44.1	100%	0.023	100%	NG_052893.1
Escherichia coli HeN867 pHeNE867 mcr-1 gene for phosphoethanolamine--lipid A transferase MCR-1.3, complete CDS	44.1	44.1	100%	0.023	100%	NG_052861.1
Escherichia coli WCHEC1606 mcr-1 gene for phosphoethanolamine--lipid A transferase MCR-1.4, complete CDS	44.1	44.1	100%	0.023	100%	NG_052664.1
Escherichia coli 1256822 mcr-1 gene for phosphoethanolamine--lipid A transferase MCR-1.5, complete CDS	44.1	44.1	100%	0.023	100%	NG_052663.1
Escherichia coli strain EC111 plasmid pEC111, complete sequence	44.1	44.1	100%	0.023	100%	KY471146.1
Escherichia coli strain EC019 plasmid pEC019, complete sequence	44.1	44.1	100%	0.023	100%	KY471145.1
Escherichia coli strain EC006 plasmid pEC006, complete sequence	44.1	44.1	100%	0.023	100%	KY471144.1
Escherichia coli strain WCHEC1604 phosphoethanolamine--lipid A transferase MCR-1.7 (mcr-1) gene, mcr-1.7 allele, complete cds	44.1	44.1	100%	0.023	100%	KY488488.1
Escherichia coli ParA (parA), hypothetical protein (pCF132_02), hypothetical protein (pCF132_03), Mcr1 (mcr1), and Pap2 (pap2) genes, complete cds	44.1	44.1	100%	0.023	100%	KY550359.1
Escherichia coli ParA (parA), hypothetical protein (pCF1-2_02), hypothetical protein (pCF1-2_03), Mcr1 (mcr1), and Pap2 (pap2) genes, complete cds	44.1	44.1	100%	0.023	100%	KY550358.1
Escherichia coli ParA (parA), hypothetical protein (pCF131_02), hypothetical protein (pCF131_03), Mcr1 (mcr1), and Pap2 (pap2) genes, complete cds	44.1	44.1	100%	0.023	100%	KY550357.1
Escherichia coli strain A434-59 plasmid phosphoethanolamine-lipid A transferase MCR-1 (mcr-1) gene, complete cds	44.1	44.1	100%	0.023	100%	KX242348.1
Escherichia coli plasmid pEc_04HAE12, complete sequence	44.1	44.1	100%	0.023	100%	KX592672.1
Salmonella Typhimurium strain HSHLJ1 plasmid pHSHLJ1-MCR1, complete sequence	44.1	44.1	100%	0.023	100%	KX856066.1
Escherichia coli strain 18HAE25 plasmid pEc_18HAE25, complete sequence	44.1	44.1	100%	0.023	100%	KY012276.1
Escherichia coli strain 27COE18 plasmid pEc_27COE18, complete sequence	44.1	44.1	100%	0.023	100%	KY012275.1
Escherichia coli strain 20COE13 plasmid pEc_20COE13, complete sequence	44.1	44.1	100%	0.023	100%	KY012274.1
Salmonella enterica subsp. enterica strain NG14043 plasmid pNG14043, complete sequence	44.1	44.1	100%	0.023	100%	KY120364.1
Salmonella enterica subsp. enterica strain C214 plasmid pC214, complete sequence	44.1	44.1	100%	0.023	100%	KY120363.1
Escherichia coli strain MRSN346355 plasmid pMRSN346355_65.5, complete sequence	44.1	44.1	100%	0.023	100%	CP018124.1
Escherichia coli strain MRSN346638 plasmid pMRSN346638_64.5, complete sequence	44.1	44.1	100%	0.023	100%	CP018118.1
Escherichia coli strain MRSN346595 plasmid pMRSN346595_64.5, complete sequence	44.1	44.1	100%	0.023	100%	CP018112.1
Escherichia coli strain MRSN352231 plasmid pMR0716_mcr1, complete sequence	44.1	44.1	100%	0.023	100%	CP018106.1
Escherichia coli nikB, mcr-1, pap2, ydfA, topB genes for relaxase, phosphoethanolamine transferase, putative PAP family transmembrane protein, hemolysin activation pr	44.1	44.1	100%	0.023	100%	LC191581.2
Klebsiella pneumoniae strain WCHKP1511 plasmid pMCR_1511, complete sequence	44.1	44.1	100%	0.023	100%	KX377410.1
Cronobacter sakazakii strain WF-5-19C plasmid pWF-5-19C_mcr-1, complete sequence	44.1	44.1	100%	0.023	100%	KX505142.1
Escherichia coli strain WF-5-19 plasmid pWF-5-19, partial sequence	44.1	44.1	100%	0.023	100%	KX505141.1
Escherichia coli strain E15017_00 plasmid pE15017_00, complete sequence	44.1	44.1	100%	0.023	100%	KX772778.1
Escherichia coli transposase (tnpA), MCR-1 (mcr-1), hypothetical protein, and transcriptional regulator (ydfA) genes, complete cds	44.1	44.1	100%	0.023	100%	KX886345.1
Salmonella enterica strain SH138 plasmid pHNSH138 sequence	44.1	44.1	100%	0.023	100%	KX257481.1
Escherichia coli strain ICBEC7P plasmid pICBEC7Pmcr, complete sequence	44.1	44.1	100%	0.023	100%	CP017246.1
Escherichia coli strain 210221272 plasmid pSLy21, complete sequence	44.1	44.1	100%	0.023	100%	CP016405.1
Escherichia coli strain GD-8 plasmid pECGD-8-33, complete sequence	44.1	44.1	100%	0.023	100%	KX254343.1
Escherichia coli strain JS-61 plasmid pECJS-61-63, complete sequence	44.1	44.1	100%	0.023	100%	KX254342.1
Escherichia coli strain JS-B60 plasmid pECJS-B60-267, complete sequence	44.1	44.1	100%	0.023	100%	KX254341.1
Escherichia coli strain NDM132 plasmid pls1 insertion sequence ISApl1 integrase (int) gene, complete cds; mcr-1 (mcr-1) and hypothetical protein genes, complete cds;	44.1	44.1	100%	0.023	100%	KX458104.1
Klebsiella pneumoniae KP-6884 unnamed5 mcr-1 gene for phosphoethanolamine--lipid A transferase MCR-1.2, complete CDS	44.1	44.1	100%	0.023	100%	NG_051170.1
Escherichia coli strain O177:H21 plasmid unnamed4, complete sequence	44.1	88.2	100%	0.023	100%	CP016550.1
Escherichia coli plasmid unnamed1, complete sequence	44.1	44.1	100%	0.023	100%	KX528699.1
Escherichia coli strain 59 plasmid pECJS-59-244, complete sequence	44.1	44.1	100%	0.023	100%	KX084394.1
Escherichia coli strain 61 plasmid pECJS-61-63, complete sequence	44.1	44.1	100%	0.023	100%	KX084393.1
Escherichia coli strain B65 plasmid pECJS-B65-33, complete sequence	44.1	44.1	100%	0.023	100%	KX084392.1
Escherichia coli strain BA76 plasmid pBA76-MCR-1, complete sequence	44.1	44.1	100%	0.023	100%	KX013540.1
Escherichia coli strain BA77 plasmid pBA77-MCR-1, complete sequence	44.1	44.1	100%	0.023	100%	KX013539.1
Escherichia coli strain ABC149 plasmid pABC149-MCR-1, complete sequence	44.1	44.1	100%	0.023	100%	KX013538.1
Kluyvera ascorbata strain WCH1410 plasmid pMCR_1410, complete sequence	44.1	44.1	100%	0.023	100%	KU922754.1
Escherichia coli strain KP81 plasmid pKP81-BE, complete sequence	44.1	44.1	100%	0.023	100%	KU994859.1
Escherichia coli plasmid pKH457-3-BE, complete sequence	44.1	44.1	100%	0.023	100%	KU353730.1
Escherichia coli strain H226B plasmid pH226B, complete sequence	44.1	44.1	100%	0.023	100%	KX129784.1
Escherichia coli strain S2.14 plasmid pS2.14-2, complete sequence	44.1	44.1	100%	0.023	100%	CP016187.1
Escherichia coli strain EC13 plasmid pEC13-1, complete sequence	44.1	44.1	100%	0.023	100%	CP016186.1
Escherichia coli strain EC5 plasmid pEC5-1, complete sequence	44.1	44.1	100%	0.023	100%	CP016185.1
Escherichia coli strain EC2_1 plasmid pEC2_1-4, complete sequence	44.1	44.1	100%	0.023	100%	CP016183.1
Escherichia coli strain FS170G plasmid pFS170G, complete sequence	44.1	44.1	100%	0.023	100%	KX711707.1
Escherichia coli strain CSZ4 plasmid pCSZ4, complete sequence	44.1	44.1	100%	0.023	100%	KX711706.1
Escherichia coli strain GD65 plasmid pGD65-4, complete sequence	44.1	44.1	100%	0.023	100%	KY075660.1
Escherichia coli strain GD80 plasmid pGD80-2, complete sequence	44.1	44.1	100%	0.023	100%	KY075659.1
Escherichia coli strain WH03 plasmid pWH03-3, complete sequence	44.1	44.1	100%	0.023	100%	KY075655.1
Escherichia coli strain Lishui12 plasmid pLishui12-2, complete sequence	44.1	44.1	100%	0.023	100%	KY075653.1
Escherichia coli strain GD46 plasmid pGD46-3, complete sequence	44.1	44.1	100%	0.023	100%	KY075652.1
Escherichia coli strain FS13Z2S plasmid pFS13Z2S, complete sequence	44.1	44.1	100%	0.023	100%	KY421937.1
Escherichia coli FS13Z2S contig 2 genomic sequence	44.1	44.1	100%	0.023	100%	KY421935.1
Escherichia coli FS13Z2S contig 1 genomic sequence	44.1	44.1	100%	0.023	100%	KY421934.1
Klebsiella pneumoniae strain KP2442 plasmid pKP2442_1c330, complete sequence	44.1	44.1	100%	0.023	100%	KX434879.1
Salmonella enterica subsp. enterica serovar Typhimurium strain YL14P053 plasmid pMCR16_P053, complete sequence	44.1	44.1	100%	0.023	100%	KY352406.1
Escherichia coli strain EHS30-1 plasmid pEHS30-1, complete sequence	44.1	44.1	100%	0.023	100%	KX772391.1
Escherichia coli strain E15004 plasmid pE15004, complete sequence	44.1	44.1	100%	0.023	100%	KX772777.1
Salmonella enterica strain Z319S plasmid pHNZ319S sequence	44.1	44.1	100%	0.023	100%	KX257482.1
Salmonella enterica strain SH36 plasmid pHNSH36 sequence	44.1	44.1	100%	0.023	100%	KX257480.1

Figure S3. *Cont.*

.

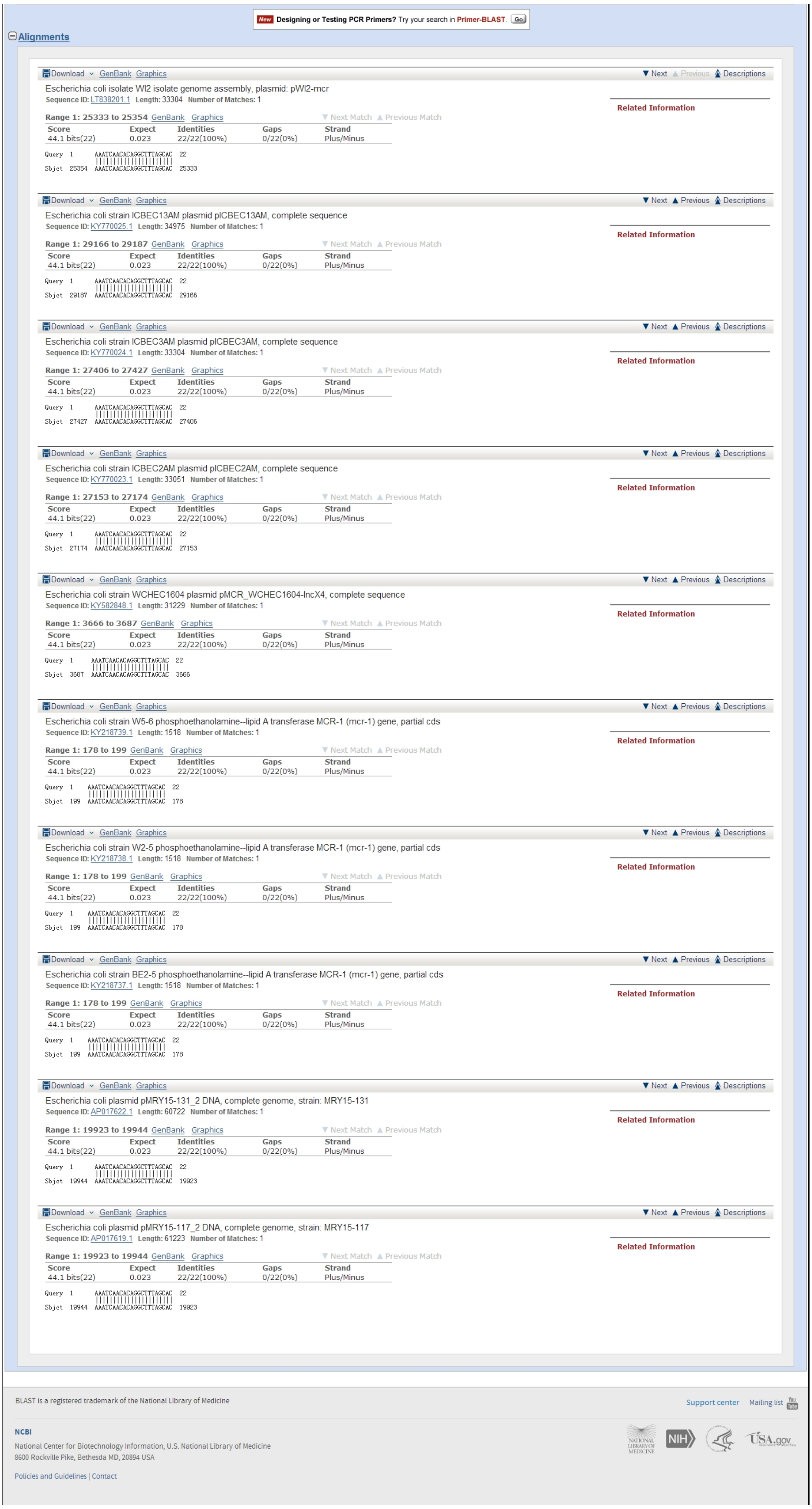

Figure S3. BLASTN analysis of primer M-R.

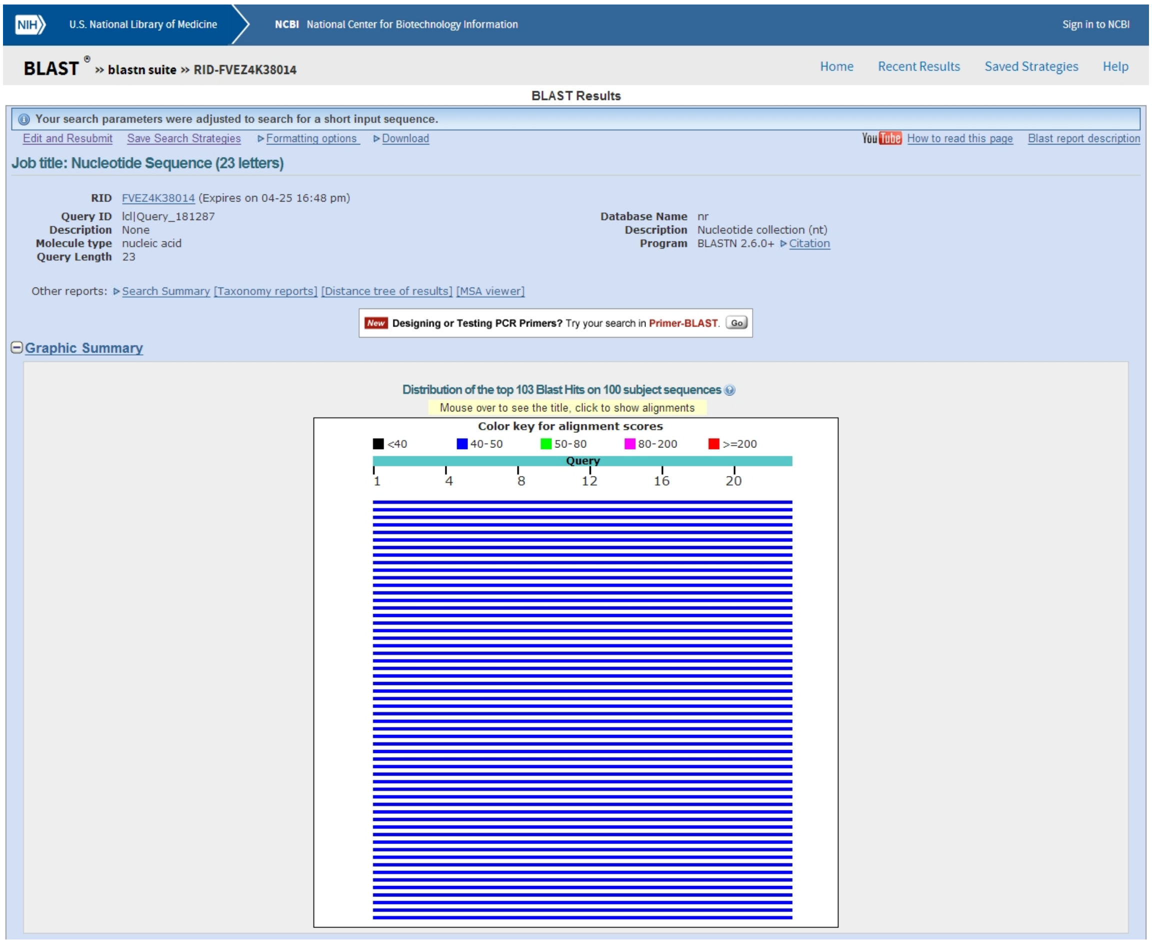

Figure S4. *Cont.*

Descriptions

Sequences producing significant alignments:

Select: All None Selected:0

Alignments Download GenBank Graphics Distance tree of results

Description	Max score	Total score	Query cover	E value	Ident	Accession
Escherichia coli isolate WI2 isolate genome assembly, plasmid: pWI2-mcr	46.1	46.1	100%	0.006	100%	LT838201.1
Escherichia coli strain ICBEC13AM plasmid pICBEC13AM, complete sequence	46.1	46.1	100%	0.006	100%	KY770025.1
Escherichia coli strain ICBEC3AM plasmid pICBEC3AM, complete sequence	46.1	46.1	100%	0.006	100%	KY770024.1
Escherichia coli strain ICBEC2AM plasmid pICBEC2AM, complete sequence	46.1	46.1	100%	0.006	100%	KY770023.1
Escherichia coli strain WCHEC1604 plasmid pMCR_WCHEC1604-IncX4, complete sequence	46.1	46.1	100%	0.006	100%	KY582848.1
Escherichia coli strain W5-6 phosphoethanolamine--lipid A transferase MCR-1 (mcr-1) gene, partial cds	46.1	46.1	100%	0.006	100%	KY218739.1
Escherichia coli strain W2-5 phosphoethanolamine--lipid A transferase MCR-1 (mcr-1) gene, partial cds	46.1	46.1	100%	0.006	100%	KY218738.1
Escherichia coli strain BE2-5 phosphoethanolamine--lipid A transferase MCR-1 (mcr-1) gene, partial cds	46.1	46.1	100%	0.006	100%	KY218737.1
Escherichia coli plasmid pMRY15-131_2 DNA, complete genome, strain: MRY15-131	46.1	46.1	100%	0.006	100%	AP017622.1
Escherichia coli plasmid pMRY15-117_2 DNA, complete genome, strain: MRY15-117	46.1	46.1	100%	0.006	100%	AP017619.1
Escherichia coli plasmid pMRY16-002_4 DNA, complete genome, strain: 20Ec-P-124	46.1	46.1	100%	0.006	100%	AP017614.1
Escherichia coli DNA, genomic structure surrounding mcr-1 gene, strain: RYU2912C-1	46.1	46.1	100%	0.006	100%	LC228070.1
Escherichia coli plasmid pKT2378 DNA, complete sequence, strain: KT2378	46.1	46.1	100%	0.006	100%	LC227558.1
Escherichia coli strain HKSH_MCR_161114268_EC phosphoethanolamine lipid A transferase (mcr1) gene, mcr1.9 allele, complete cds	46.1	46.1	100%	0.006	100%	KY685071.1
Escherichia coli strain HKSH_MCR_161103180_EC phosphoethanolamine lipid A transferase (mcr1) gene, mcr1.8 allele, complete cds	46.1	46.1	100%	0.006	100%	KY685070.1
Escherichia coli strain pPY1 plasmid pPY1, complete sequence	46.1	46.1	100%	0.006	100%	KX711708.1
Escherichia coli strain FS170G plasmid pFS170G, complete sequence	46.1	46.1	100%	0.006	100%	KX711707.1
Escherichia coli strain CSZ4 plasmid pCSZ4, complete sequence	46.1	46.1	100%	0.006	100%	KX711706.1
Escherichia coli strain EC590, complete genome	46.1	138	100%	0.006	100%	CP016182.2
Citrobacter braakii strain CA-26 plasmid pCA-26, complete sequence	46.1	46.1	100%	0.006	100%	KY624633.1
Escherichia coli strain GD53 plasmid pGD53-3, complete sequence	46.1	46.1	100%	0.006	100%	KY075662.1
Escherichia coli strain GD65 plasmid pGD65-3, complete sequence	46.1	46.1	100%	0.006	100%	KY075661.1
Escherichia coli strain GD65 plasmid pGD65-4, complete sequence	46.1	46.1	100%	0.006	100%	KY075660.1
Escherichia coli strain GD80 plasmid pGD80-2, complete sequence	46.1	46.1	100%	0.006	100%	KY075659.1
Escherichia coli strain WH07 plasmid pWH07-3, complete sequence	46.1	46.1	100%	0.006	100%	KY075658.1
Escherichia coli strain WH09 plasmid pWH09-3, complete sequence	46.1	46.1	100%	0.006	100%	KY075657.1
Escherichia coli strain WH13 plasmid pWH13-4, complete sequence	46.1	46.1	100%	0.006	100%	KY075656.1
Escherichia coli strain WH03 plasmid pWH03-3, complete sequence	46.1	46.1	100%	0.006	100%	KY075655.1
Escherichia coli strain Lishui142 plasmid pLishui142-1, complete sequence	46.1	46.1	100%	0.006	100%	KY075654.1
Escherichia coli strain Lishui12 plasmid pLishui12-2, complete sequence	46.1	46.1	100%	0.006	100%	KY075653.1
Escherichia coli strain GD46 plasmid pGD46-3, complete sequence	46.1	46.1	100%	0.006	100%	KY075652.1
Escherichia coli strain GD23 plasmid pGD23-3, complete sequence	46.1	46.1	100%	0.006	100%	KY075651.1
Escherichia coli strain GD17 plasmid pGD17-2, complete sequence	46.1	46.1	100%	0.006	100%	KY075650.1
Escherichia coli strain FS13Z2S plasmid pFS13Z2S, complete sequence	46.1	46.1	100%	0.006	100%	KY421937.1
Escherichia coli FS13Z2S contig 3 genomic sequence	46.1	46.1	100%	0.006	100%	KY421936.1
Escherichia coli FS13Z2S contig 2 genomic sequence	46.1	46.1	100%	0.006	100%	KY421935.1
Escherichia coli FS13Z2S contig 1 genomic sequence	46.1	46.1	100%	0.006	100%	KY421934.1
Escherichia coli strain ST101 phosphoethanolamine transferase (mcr-1.8) gene, complete cds	46.1	46.1	100%	0.006	100%	KY683842.1
Escherichia coli strain C2-007R phosphoethanolamine--lipid A transferase (mcr-1) gene, partial cds	46.1	46.1	100%	0.006	100%	KY013597.1
Escherichia coli strain MDR_56 plasmid pMCR1-NY, complete sequence	46.1	46.1	100%	0.006	100%	CP019908.1
Cloning vector pGDP2, complete sequence	46.1	46.1	100%	0.006	100%	KX859085.1
Salmonella enterica subsp. enterica serovar Typhimurium YL14P053 pMCR16_P053 mcr-1 gene for phosphoethanolamine--lipid A transferase MCR-1.6, complete CDS	46.1	46.1	100%	0.006	100%	NG_052893.1
Escherichia coli HeN867 pHeNE867 mcr-1 gene for phosphoethanolamine--lipid A transferase MCR-1.3, complete CDS	46.1	46.1	100%	0.006	100%	NG_052861.1
Escherichia coli WCHEC1606 mcr-1 gene for phosphoethanolamine--lipid A transferase MCR-1.4, complete CDS	46.1	46.1	100%	0.006	100%	NG_052664.1
Escherichia coli 1256822 mcr-1 gene for phosphoethanolamine--lipid A transferase MCR-1.5, complete CDS	46.1	46.1	100%	0.006	100%	NG_052663.1
Escherichia coli strain EC111 plasmid pEC111, complete sequence	46.1	46.1	100%	0.006	100%	KY471146.1
Escherichia coli strain EC019 plasmid pEC019, complete sequence	46.1	46.1	100%	0.006	100%	KY471145.1
Escherichia coli strain EC006 plasmid pEC006, complete sequence	46.1	46.1	100%	0.006	100%	KY471144.1
Escherichia coli strain WCHEC1604 phosphoethanolamine--lipid A transferase MCR-1.7 (mcr-1) gene, mcr-1.7 allele, complete cds	46.1	46.1	100%	0.006	100%	KY488488.1
Klebsiella pneumoniae strain KP2442 plasmid pKP2442_1c330, complete sequence	46.1	46.1	100%	0.006	100%	KX434879.1
Escherichia coli ParA (parA), hypothetical protein (pCF132_02), hypothetical protein (pCF132_03), Mcr1 (mcr1), and Pap2 (pap2) genes, complete cds	46.1	46.1	100%	0.006	100%	KY550359.1
Escherichia coli ParA (parA), hypothetical protein (pCF1-2_02), hypothetical protein (pCF1-2_03), Mcr1 (mcr1), and Pap2 (pap2) genes, complete cds	46.1	46.1	100%	0.006	100%	KY550358.1
Escherichia coli ParA (parA), hypothetical protein (pCF131_02), hypothetical protein (pCF131_03), Mcr1 (mcr1), and Pap2 (pap2) genes, complete cds	46.1	46.1	100%	0.006	100%	KY550357.1
Escherichia coli strain A434-59 plasmid phosphoethanolamine-lipid A transferase MCR-1 (mcr-1) gene, complete cds	46.1	46.1	100%	0.006	100%	KX242348.1
Escherichia coli plasmid pEc_04HAE12, complete sequence	46.1	46.1	100%	0.006	100%	KX592672.1
Salmonella enterica subsp. enterica serovar Typhimurium strain YL14P053 plasmid pMCR16_P053, complete sequence	46.1	46.1	100%	0.006	100%	KY352406.1
Salmonella Typhimurium strain HSHLJ1 plasmid pHSHLJ1-MCR1, complete sequence	46.1	46.1	100%	0.006	100%	KX856066.1
Escherichia coli strain 18HAE25 plasmid pEc_18HAE25, complete sequence	46.1	46.1	100%	0.006	100%	KY012276.1
Escherichia coli strain 27COE18 plasmid pEc_27COE18, complete sequence	46.1	46.1	100%	0.006	100%	KY012275.1
Escherichia coli strain 20COE13 plasmid pEc_20COE13, complete sequence	46.1	46.1	100%	0.006	100%	KY012274.1
Salmonella enterica subsp. enterica strain R150626 plasmid pR150626, complete sequence	46.1	46.1	100%	0.006	100%	KY120366.1
Salmonella enterica subsp. enterica strain P111 plasmid pP111, complete sequence	46.1	46.1	100%	0.006	100%	KY120365.1
Salmonella enterica subsp. enterica strain NG14043 plasmid pNG14043, complete sequence	46.1	46.1	100%	0.006	100%	KY120364.1
Salmonella enterica subsp. enterica strain C214 plasmid pC214, complete sequence	46.1	46.1	100%	0.006	100%	KY120363.1
Escherichia coli strain MRSN346355 plasmid pMRSN346355_65.5, complete sequence	46.1	46.1	100%	0.006	100%	CP018124.1
Escherichia coli strain MRSN346638 plasmid pMRSN346638_64.5, complete sequence	46.1	46.1	100%	0.006	100%	CP018118.1
Escherichia coli strain MRSN346595 plasmid pMRSN346595_64.5, complete sequence	46.1	46.1	100%	0.006	100%	CP018112.1
Escherichia coli strain MRSN352231 plasmid pMR0716_mcr1, complete sequence	46.1	46.1	100%	0.006	100%	CP018106.1
Escherichia coli strain EHS30-1 plasmid pEHS30-1, complete sequence	46.1	46.1	100%	0.006	100%	KX772391.1
Escherichia coli nikB, mcr-1, pap2, ydfA, topB genes for relaxase, phosphoethanolamine transferase, putative PAP family transmembrane protein, hemolysin activation pr	46.1	46.1	100%	0.006	100%	LC191581.2
Klebsiella pneumoniae strain WCHKP1511 plasmid pMCR_1511, complete sequence	46.1	46.1	100%	0.006	100%	KX377410.1
Cronobacter sakazakii strain WF-5-19C plasmid pWF-5-19C_mcr-1, complete sequence	46.1	46.1	100%	0.006	100%	KX505142.1
Escherichia coli strain WF-5-19 plasmid pWF-5-19, partial sequence	46.1	46.1	100%	0.006	100%	KX505141.1
Escherichia coli strain E15017_00 plasmid pE15017_00, complete sequence	46.1	46.1	100%	0.006	100%	KX772778.1
Escherichia coli strain E15004 plasmid pE15004, complete sequence	46.1	46.1	100%	0.006	100%	KX772777.1
Escherichia coli transposase (tnpA), MCR-1 (mcr-1), hypothetical protein, and transcriptional regulator (ydfA) genes, complete cds	46.1	46.1	100%	0.006	100%	KX886345.1
Salmonella enterica strain Z319S plasmid pHNZ319S sequence	46.1	46.1	100%	0.006	100%	KX257482.1
Salmonella enterica strain SH138 plasmid pHNSH138 sequence	46.1	46.1	100%	0.006	100%	KX257481.1
Salmonella enterica strain SH36 plasmid pHNSH36 sequence	46.1	46.1	100%	0.006	100%	KX257480.1
Escherichia coli strain ICBEC7P plasmid pICBEC7Pmcr, complete sequence	46.1	46.1	100%	0.006	100%	CP017246.1
Escherichia coli strain 210221272 plasmid pSLy21, complete sequence	46.1	46.1	100%	0.006	100%	CP016405.1
Escherichia coli strain CQ02-121 plasmid pCQ02-121, complete sequence	46.1	46.1	100%	0.006	100%	KU647721.2
Escherichia coli strain GD-8 plasmid pECGD-8-33, complete sequence	46.1	46.1	100%	0.006	100%	KX254343.1
Escherichia coli strain JS-61 plasmid pECJS-61-63, complete sequence	46.1	46.1	100%	0.006	100%	KX254342.1
Escherichia coli strain JS-B60 plasmid pECJS-B60-267, complete sequence	46.1	46.1	100%	0.006	100%	KX254341.1
Escherichia coli strain NDM132 plasmid pls1 insertion sequence ISApl1 integrase (int) gene, complete cds; mcr-1 (mcr-1) and hypothetical protein genes, complete cds;	46.1	46.1	100%	0.006	100%	KX458104.1
Escherichia coli strain MCR1_NJ plasmid pMCR1-NJ-IncX4, complete sequence	46.1	46.1	100%	0.006	100%	KX447768.1
Klebsiella pneumoniae KP-6884 unnamed5 mcr-1 gene for phosphoethanolamine--lipid A transferase MCR-1.2, complete CDS	46.1	46.1	100%	0.006	100%	NG_051170.1
Escherichia coli strain O177:H21 plasmid unnamed4, complete sequence	46.1	92.2	100%	0.006	100%	CP016550.1
Escherichia coli plasmid unnamed1, complete sequence	46.1	46.1	100%	0.006	100%	KX528699.1
Escherichia coli strain 59 plasmid pECJS-59-244, complete sequence	46.1	46.1	100%	0.006	100%	KX084394.1
Escherichia coli strain 61 plasmid pECJS-61-63, complete sequence	46.1	46.1	100%	0.006	100%	KX084393.1
Escherichia coli strain B65 plasmid pECJS-B65-33, complete sequence	46.1	46.1	100%	0.006	100%	KX084392.1
Escherichia coli strain BA76 plasmid pBA76-MCR-1, complete sequence	46.1	46.1	100%	0.006	100%	KX013540.1
Escherichia coli strain BA77 plasmid pBA77-MCR-1, complete sequence	46.1	46.1	100%	0.006	100%	KX013539.1
Escherichia coli strain ABC149 plasmid pABC149-MCR-1, complete sequence	46.1	46.1	100%	0.006	100%	KX013538.1
Kluyvera ascorbata strain WCH1410 plasmid pMCR_1410, complete sequence	46.1	46.1	100%	0.006	100%	KU922754.1
Escherichia coli strain KP81 plasmid pKP81-BE, complete sequence	46.1	46.1	100%	0.006	100%	KU994859.1
Escherichia coli plasmid pKH457-3-BE, complete sequence	46.1	46.1	100%	0.006	100%	KU353730.1
Escherichia coli strain H226B plasmid pH226B, complete sequence	46.1	46.1	100%	0.006	100%	KX129784.1

Figure S4. *Cont.*

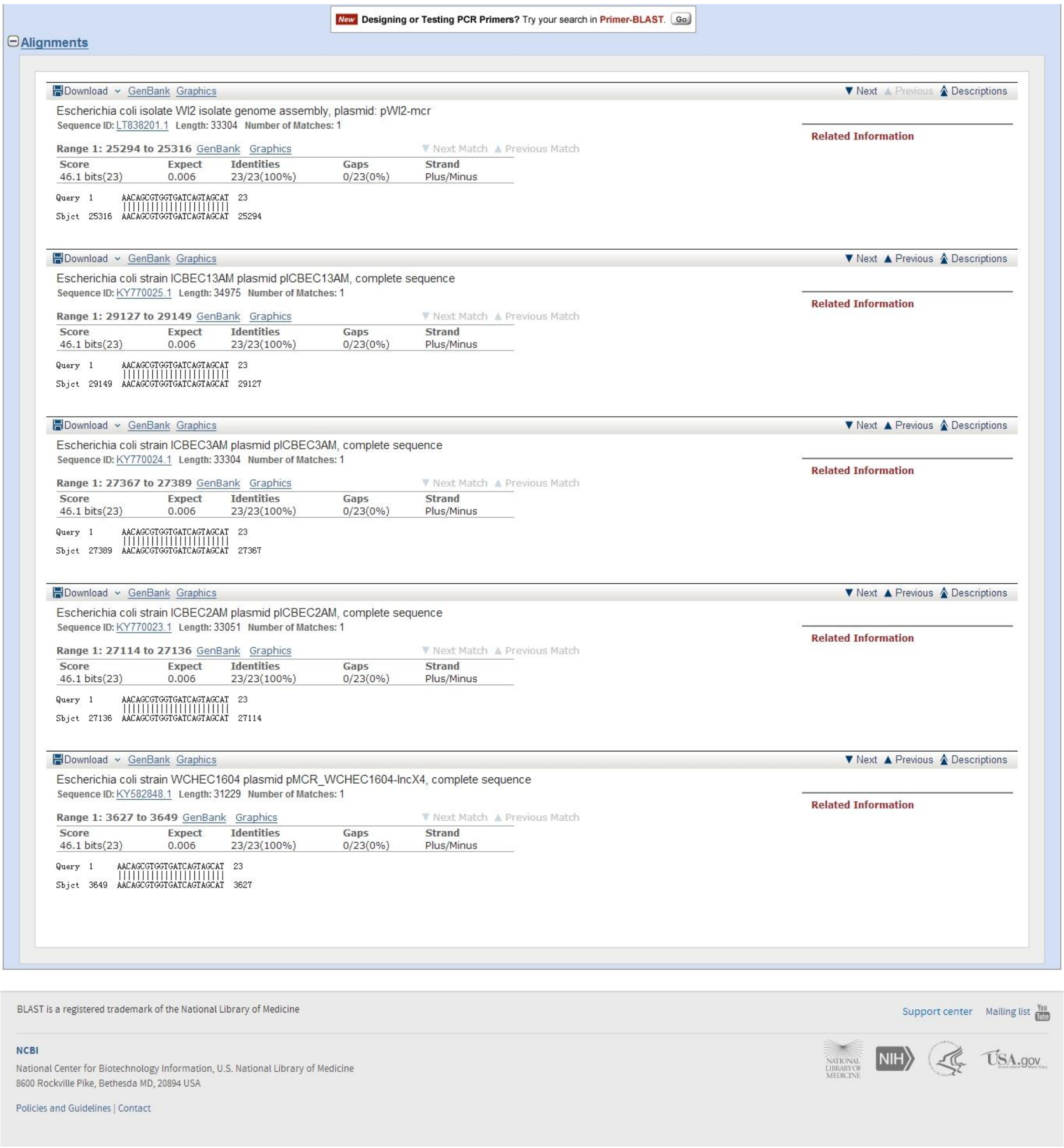

Figure S4. BLASTN analysis of primer M-Probe.

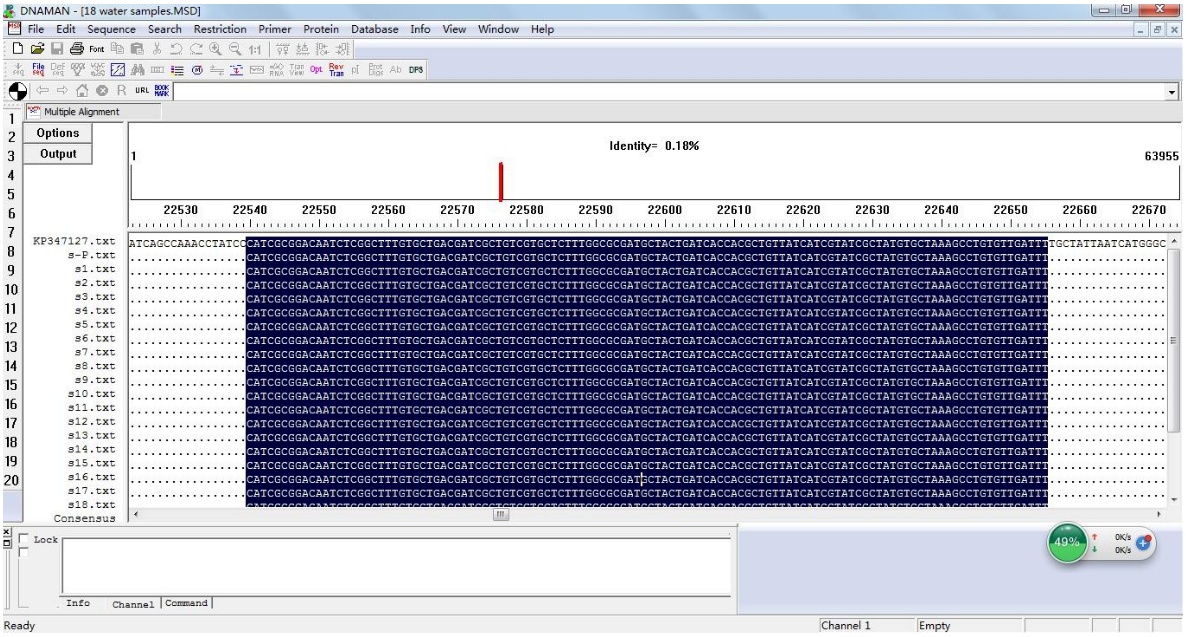

Figure S5. Alignment of the sequences of qPCR products and the *mcr-1* gene sequence: (KP347127: the *mcr-1* gene sequence reported by Liu and colleagues, s-P: positive control, s-1–s-18: water samples).

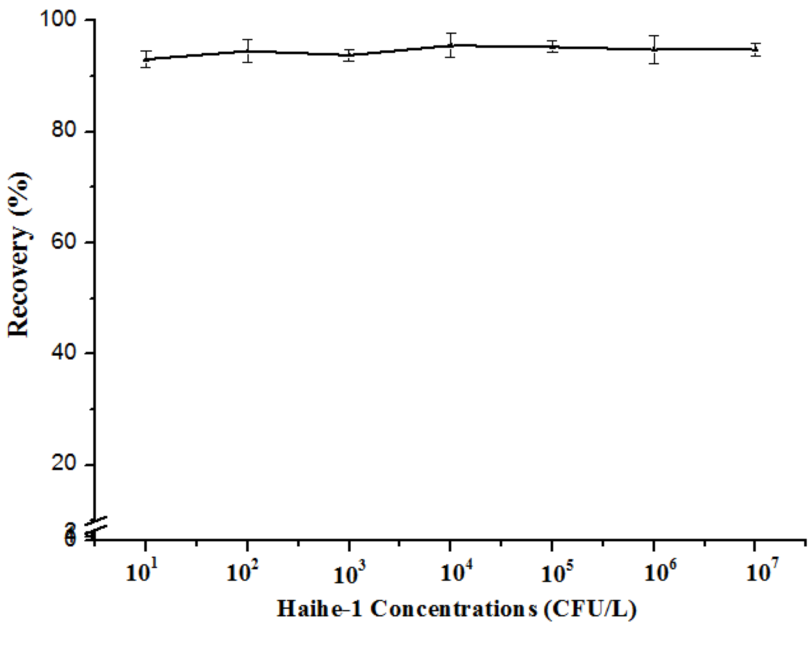

Figure S6. Recovery of Haihe-1 strain (10^1 CFU/L–10^7 CFU/L) spiked into 10L PBS

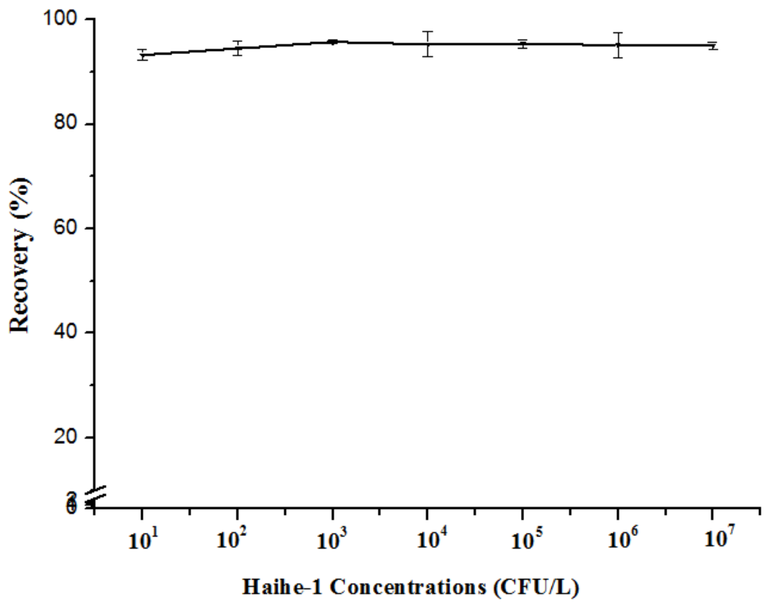

Figure S7. Recovery of Haihe-1 strain (10^1CFU/L–10^7CFU/L) spiked into 10L sterilized waster sample.

Univariate Analysis of Variance

Between-Subjects Factors

		N
site	HD	6
	HE	6
	HU	6
time	Apr	3
	Feb	3
	Jan	3
	June	3
	Mar	3
	May	3

Tests of Between-Subjects Effects

Dependent Variable: lgGC

Source	Type III Sum of Squares	df	Mean Square	F	Sig.
Corrected Model	3.920[a]	7	.560	6.287	.005
Intercept	339.259	1	339.259	3808.794	.000
site	2.576	2	1.288	14.459	.001
time	1.344	5	.269	3.019	.065
Error	.891	10	.089		
Total	344.070	18			
Corrected Total	4.811	17			

a. R Squared = .815 (Adjusted R Squared = .685)

Figure S8. The multiple comparisons result of ANOVA.

Post Hoc Tests

site

Multiple Comparisons

Dependent Variable: lgGC

Bonferroni

(I) site	(J) site	Mean Difference (I-J)	Std. Error	Sig.	95% Confidence Interval	
					Lower Bound	Upper Bound
HD	HE	-.644917*	.1723104	.011	-1.139460	-.150373
	HU	.253731	.1723104	.515	-.240812	.748275
HE	HD	.644917*	.1723104	.011	.150373	1.139460
	HU	.898648*	.1723104	.001	.404105	1.393192
HU	HD	-.253731	.1723104	.515	-.748275	.240812
	HE	-.898648*	.1723104	.001	-1.393192	-.404105

Based on observed means.
The error term is Mean Square(Error) = .089.

*. The mean difference is significant at the 0.05 level.

Figure S9. The deviance analysis result of ANOVA.

© 2017 by the authors; licensee MDPI, Basel, Switzerland. This article is an open access article distributed under the terms and conditions of the Creative Commons by Attribution (CC-BY) license (http://creativecommons.org/licenses/by/4.0/)
